# Three New Heptelidic Acid Derivatives from the Culture of Mushroom *Lentinellus ursinus*

**DOI:** 10.1007/s13659-018-0168-8

**Published:** 2018-05-22

**Authors:** Li Liu, Jun-Jie Han, Tian-Shun Xu, Rui-Xing Liu, Li Bao, Hong-Wei Liu

**Affiliations:** 1 State Key Laboratory of Mycology, Institute of Microbiology, Chinese Academy of Sciences, Beijing, 100101 People’s Republic of China; 2 Savaid Medical School, University of Chinese Academy of Sciences, Beijing, 100049 People’s Republic of China; 3 College of Life Sciences, Hebei University, Baoding, 071002 China

**Keywords:** *Lentinellus ursinus*, Heptelidic acid derivatives, Biosynthetic pathway

## Abstract

**Abstract:**

Three new heptelidic acid derivatives (**1**–**3**) including two new dimeric esters and two known heptelidic acid analogues (**4** and **5**) were isolated from the solid culture of mushroom *Lentinellus ursinus*. The structures of new compounds were confirmed by the analysis of NMR and HRESIMS spectroscopic data. The biosynthetic origin of compounds **1**–**5** was postulated. Compounds **1**–**5** exhibited no antibacterial activity against *Staphylococcus aureus* and *Escherichia coli* at the dose of 100 μM.

**Graphical Abstract:**

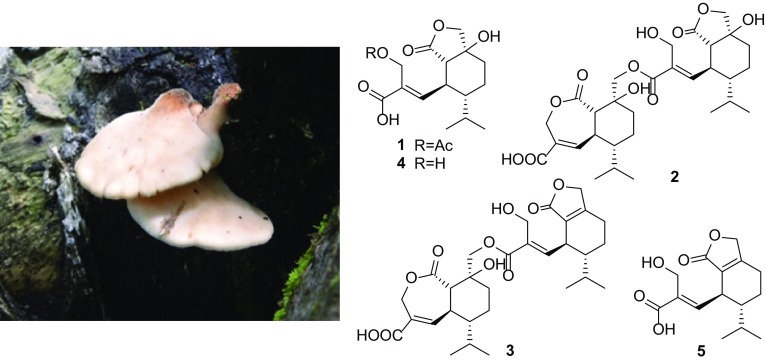

**Electronic supplementary material:**

The online version of this article (10.1007/s13659-018-0168-8) contains supplementary material, which is available to authorized users.

## Introduction

The mushrooms in the genus of *Lentinellus* are white rot, wood decay, and characterized with rough-walled and amyloid spores. Eighteen species and varieties of *Lentinellus* have been described all over the world. There have been eleven species reported in China [1, 2].

**Fig. 1:**
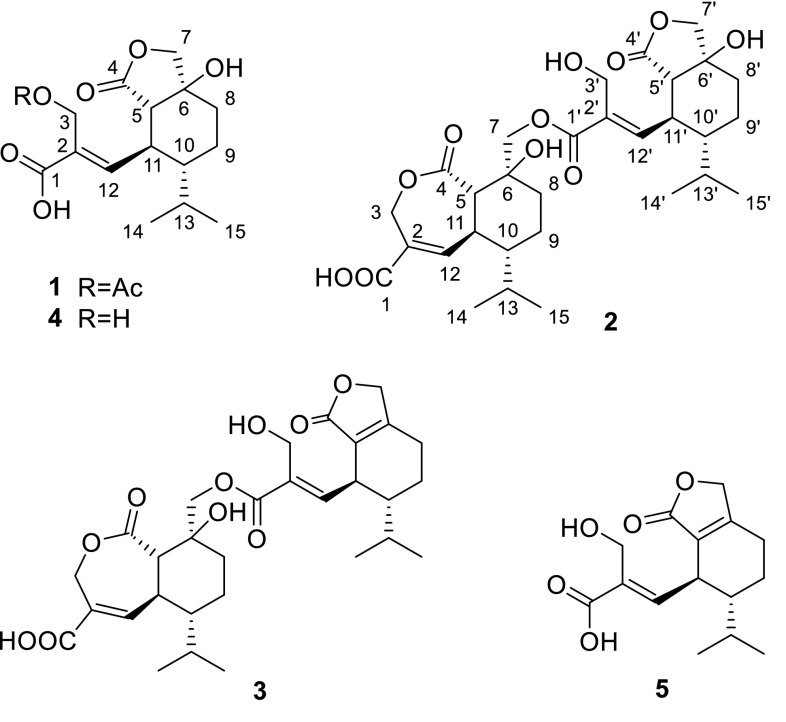
The structures of compounds **1**–**5** from *L. ursinus*

Mushroom-derived natural products draw much attention of chemists and biologists due to their diverse structural skeletons and interesting biological activities [3]. Mushrooms have been known as a prolific source of structurally diverse sesquiterpenes [4–7]. Heptelidic acid and its analogues are a group of significant secondary metabolites with interesting biological activities, such as cytotoxic, antimicrobial, antimalarial activities [8–10]. So far, heptelidic acid and its derivatives have been reported from the genus of fungi *Lentinellus*, *Gliocladium*, *Chaetomium*, *Trichoderma*, *Xylaria*, *Phyllosticta*, and *Acremonium* [8, 9, 11–13]. In our ongoing search for new sesquiterpenes from mushrooms, the EtOAc extract from the solid culture of *Lentinellus ursinus* was investigated. To date, only one sesquiterpene lentinellic acid was reported from the liquid culture of *L. ursinus* [14]. In this study, five heptelidic acid derivatives including a new acetylated compound and two new dimeric sesquiterpenoid esters were isolated from the fungus *L. ursinus*. Herein, we report the isolation, structure elucidation, and antibacterial activity evaluation of new compounds **1**–**3** (Fig. 1).

## Results and Discussion

The fungus *L. ursinus* was fermented on rice medium. The EtOAc extract of its rice culture was subjected to silica gel, ODS, Sephadex LH-20, and HPLC chromatography to afford three new compounds 3-*O*-acetylheptelidic acid A (**1**) lentisinic acid A (**2**) and B (**3**), and two known compounds hydroheptelidic acid (**4**) [12] and xylaric acid D (**5**) [11].

**Fig. 2:**
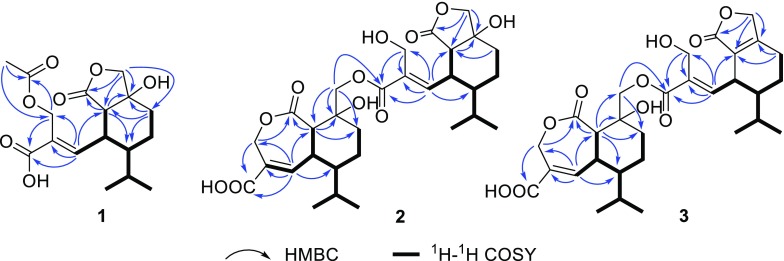
Key HMBC and ^1^H–^1^H COSY correlations of compounds **1**–**3**

**Fig. 3:**
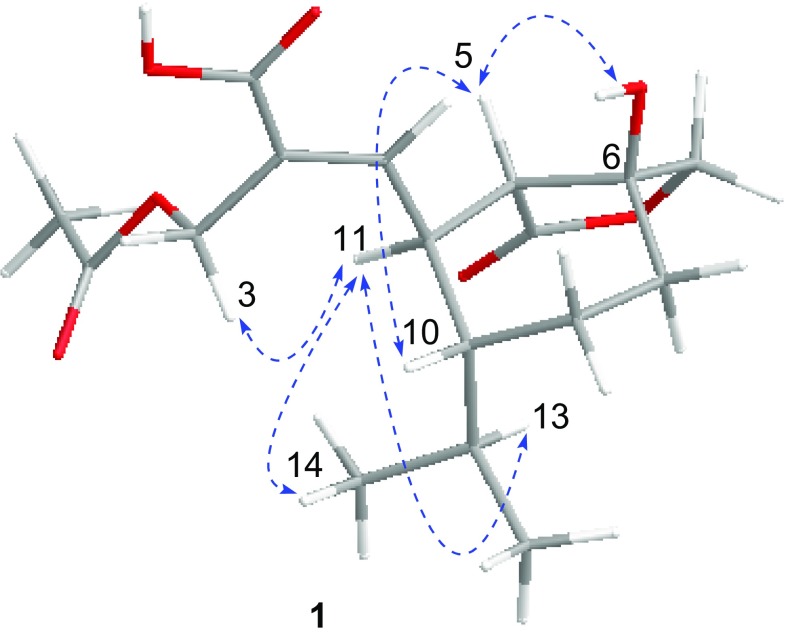
Key NOE correlations of compound **1**

3-*O*-Acetylheptelidic acid A (**1**) was obtained as a colorless oil. Its molecular formula was determined to be C_17_H_24_O_7_ by the HRESIMS (*m/z* 363.1414 [M+Na]^+^). The ^1^H, ^13^C NMR, and HSQC data (Table 1) displayed a singlet methyl (*δ*_C_/*δ*_H_ 20.9/2.00), two doublet methyls [*δ*_C_/*δ*_H_ 15.9/0.75 (d, *J* = 6.9 Hz), 21.9/0.97 (d, *J* = 6.9 Hz)], four methylenes containing two oxygenated methylenes [*δ*_C_/*δ*_H_ 59.2/4.82 (d, *J* = 12.1 Hz), 4.59 (d, *J* = 12.1 Hz); 76.2/4.45 (d, *J* = 9.9 Hz), 4.06 (d, *J* = 9.9 Hz)], four methines, a trisubstituted double bond (*δ*_C_/*δ*_H_ 148.8/6.80 (d, *J* = 9.9 Hz), 130.8), an oxygenated quaternary carbon (*δ*_C_ 76.3), and three carbonyl carbons (*δ*_C_ 169.2, 172.5, 178.8). These data indicated that **1** shares the same sesquiterpenoid skeleton with hydroheptelidic acid (**4**) [12], except for an additional acetyl group. The ^1^H–^1^H COSY correlations of H-11/H-12, H-5/H-11/H-10/H_2_-9/H_2_-8, H-10/H-13/H_3_-14(15), and key HMBC correlations of H_2_-3 with C-1 and C-2, H-12 with C-1, C-2, C-3, and C-5, H-5 with C-6 and C-4, H_2_-7 with C-4, C-6, and C-5, H-8 with C-5, C-6 and C-7, H_2_-3 (*δ*_H_ 4.82, 4.59) and the methyl group (*δ*_H_ 2.00) with the carbonyl carbon (*δ*_C_ 172.5) indicated the attachment of the acetyl group at C-3 definitely assigned the structure of **1** (Fig. 2). The strong NOE correlations of H-11 with H_2_-3 supported the *E*-configuration for the C(2)=C(12) bond. The NOE correlations of H-11 with H-13, H_3_-14 and H_3_-15, H-5 with H-10, together with the larger coupling constants (^3^*J*_5, 11_ = 11.0 Hz) between H-5 and H-11 attributed the *a*-orientation for H-11 and *β*-orientation for H-5 and H-10 (Fig. 3). The obvious NOE correlation of HO-6 (*δ*_H_ 5.38 in DMSO-*d*_6_) with H-6 (*δ*_H_ 2.06 in DMSO-*d*_6_) assigned the *β* configuration of HO-6. Considering the same biosynthetic pathway for the heptelidic acid derivatives, the absolute configuration of **1** was deduced to be 5*S*, 6*S*, 10*R*, 11*S*.

**Table 1:** ^1^H NMR and ^13^C NMR spectroscopic data of compounds **1**, **4** and **5** in CD_3_OD

Position	**1**		**4**		**5**	
	*δ*_C_	*δ*_H_ (mult, *J* in Hz)	*δ*_C_	*δ*_H_ (mult, *J* in Hz)	*δ*_C_	*δ*_H_ (mult, *J* in Hz)
1	169.2		170.1		170.1	
2	130.8		124.9		125.9	
3	59.2	4.82 d (12.1) 4.59 d (12.1)	57.4	4.13 d (12.3) 4.27 d (12.3)	57.4	4.45 s
4	178.8		179.4		176.1	
5	53.8	2.13 d (11.0)	53.9	2.12 d (10.9)	135.3	
6	76.3		76.4		167.2	
7	76.2	4.45 d (9.9) 4.06 d (9.9)	76.2	4.48 d (9.9) 4.08 d (9.9)	73.4	4.81 d (12.5)
8	32.9	2.03 m^a^, 1.68 m	32.9	2.03 m, 1.69 m	23.8	2.45 m
9	22.0	1.78 m, 1.26 m	22.0	1.79 m, 1.29 m	22.0	1.94 m, 1.59 m
10	46.4	1.36 m	46.6	1.35 m	46.8	1.52 m
11	42.0	2.62 m	41.6	2.69 m	35.7	3.54 m
12	148.8	6.80 d (10.9)	145.5	6.64 d (10.6)	146.0	6.51 d (10.8)
13	29.5	1.72 m	29.5		29.0	
14	15.9	0.75 d (6.9)	15.8	0.79 d (6.9)	17.9	0.90 d (6.8)
15	21.9	0.97 d (6.9)	21.9	0.99 d (6.9)	21.4	1.01 d (6.8)
16	172.5					
17	20.9	2.00 s				

^a^“m” means multiplet with other signals

**Fig. 4:**
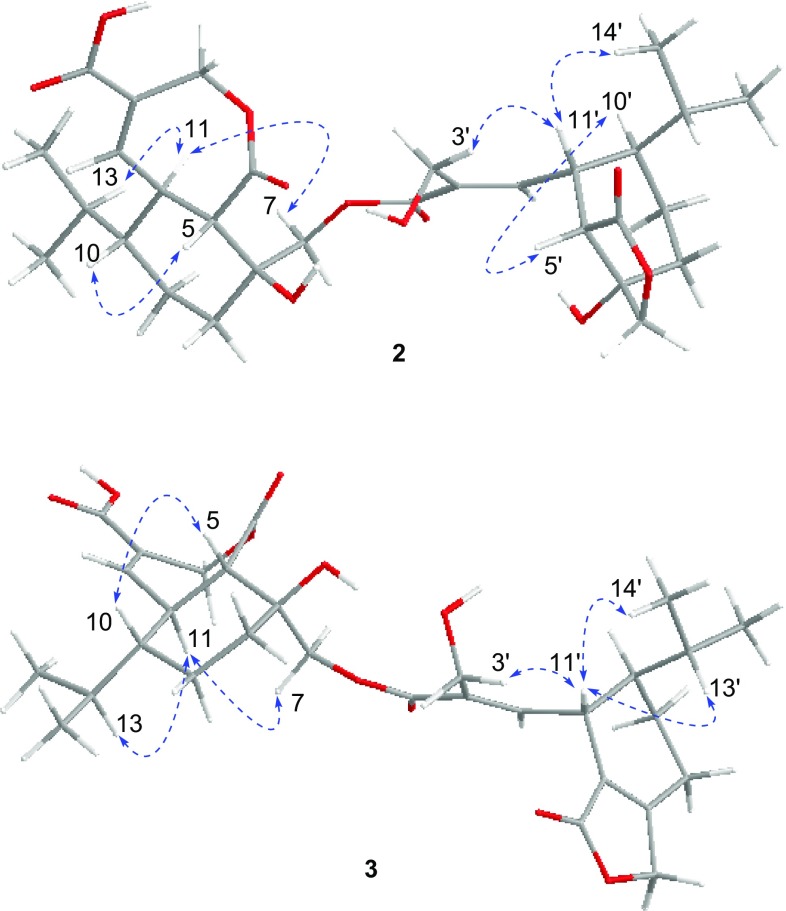
Key NOE correlations of compound **2** and **3**

The molecular formula of **2** was determined to be C_30_H_42_O_11_ with 10 degrees of unsaturation, as determined by HRESIMS (*m/z* [M+Na]^+^ 601.2615). The ^1^H, ^13^C NMR, and HSQC data of **2** revealed the presence of four doublet methyls [*δ*_C_/*δ*_H_ 15.6/0.94 (d, *J* = 6.9 Hz), 15.9/0.79 (d, *J* = 6.9 Hz), 21.6/1.00 (d, *J* = 6.9 Hz), 21.9/0.98 (d, *J* = 6.9 Hz)], eight methylenes containing four oxygenated methylenes [*δ*_C_/*δ*_H_ 57.2/4.30 (d, *J* = 12.4 Hz), 4.16 (d, *J* = 12.4 Hz); 62.7/5.23 (d, *J* = 14.6 Hz), 5.06 (d, *J* = 14.6 Hz); 66.3/5.18 (d, *J* = 12.3 Hz), 4.59 (d, *J* = 12.3 Hz); 76.3/4.48 (d, *J* = 9.9 Hz), 4.08 (d, *J* = 9.9 Hz)], eight methines, two pairs of trisubstituted olefinic carbons [*δ*_C_/*δ*_H_ 147.0/7.28 (d, *J* = 4.6 Hz), 130.9; 146.1/6.71 (d, *J* = 10.7 Hz), 134.7], two oxygenated quaternary carbon (*δ*_C_ 74.0, 76.4), and four carbonyl carbons (*δ*_C_ 168.4, 174.4, 168.5, 179.5) (Table 2). The HMBC and ^1^H–^1^H COSY spectral analysis (Fig. 2) confirmed the presence of two sesquiterpenes moieties, respectively, corresponding to heptelidic acid [15] and hydroheptelidic acid (**4**) [12]. The heptelidic acid residue was determined by ^1^H–^1^H COSY correlations of H-11/H-12, H-5/H-11/H-10/H_2_-9/H_2_-8, H-10/H-13/H_3_-14(15), and key HMBC correlations from H_2_-3 to C-1 and C-4, H-5 to C-4 and C-6, H_2_-7 to C-5, C-6 and C-8, H-12 to C-1, C-2, C-3, C-5 and C-10. The remaining signals were belonging to the hydroheptelidic acid moiety was assigned by 2D NMR spectral data. Finally, an ester bond was assigned between C-7 and C-1′ to satisfy the requirement of the molecular weight, which was also supported by the HMBC correlations of H-7 (*δ*_H_ 5.18, 4.59) with C-1′ (*δ*_C_ 168.4). NOE correlations of H-5/H-10, H-11/H_2_-7, H-11/H-13, H-3′/H-11′, H-11′/H_3_-14′, H-5′/H-10′, together with the lager coupling constants of C-5 and C-11 (^3^*J*_5,11_ = 12.6 Hz), H-5′ and H-11′ (^3^*J*_5′,11′_ = 10.5 Hz) confirmed the relative configuration in **2** as described (Fig. 4).

**Table 2:** ^1^H NMR and ^13^C NMR spectroscopic data of compounds **2** and **3**

Position	**2**		**3**	
	*δ*_C_	*δ*_H_ (mult, *J* in Hz)	*δ*_C_	*δ*_H_ (mult, *J* in Hz)
1	168.4		168.5	
2	130.9		130.1	
3	62.7	5.23 d (14.6), 5.06 d (14.6)	61.7	4.98 o^b^
4	174.4		172.7	
5	53.8	3.64 d (12.6)	54.1	3.14 d (12.7)
6	74.0		73.4	
7	66.3	5.18 d (12.3), 4.59 d (12.3)	66.3	4.99 o, 4.37 d (12.1)
8	36.0	2.32 dt (12.3, 3.2), 1.43 m	35.4	2.14 d (12.9), 1.48 m
9	22.1	1.76 m, 1.40 m^a^	20.9	1.74 m, 1.32 m
10	49.6	1.60 m	48.1	1.50 m
11	41.7	2.68 m	39.1	2.66 m
12	147.0	7.28 d (4.5)	150.2	7.31 d (5.0)
13	28.7	2.13 m	27.9	2.02 m
14	15.6	0.94 d (6.9)	15.4	0.84 d (6.8)
15	21.6	1.00 d (6.9)	21.4	0.97 d (6.8)
1′	168.4		167.1	
2′	134.7		133.4	
3′	57.2	4.30 d (12.4), 4.16 d (12.4)	56.9	4.53 s
4′	179.5		174.5	
5′	54.0	2.14 d (10.5)	125.3	
6′	76.4		164.7	
7′	76.3	4.48 d (9.9), 4.08 d (9.9)	72.2	4.82 d (17.5), 4.73 d (17.5)
8′	32.9	2.03 dt (14.0, 3.7), 1.68 dd (14.0, 4.6)	23.1	2.41 m
9′	22.3	1.78 m, 1.29 m	20.5	2.00 m, 1.63 m
10′	46.6	1.38 m	45.1	1.54 m
11′	41.1	2.70 m	34.3	3.56 m
12′	146.1	6.71 d (10.7)	145.2	6.56 d (10.8)
13′	29.6	1.79 m	28.3	1.64 m
14′	15.9	0.79 d (6.9)	18.0	0.88 d (6.7)
15′	21.9	0.98 d (6.9)	21.8	1.00 d (6.7)

Recorded in CD_3_OD

Recorded in CDCl_3_

^a^“m” means multiplet with other signals

^b^“o” means overlapped with other signals

Lentisinic acid B (**3**) has the molecular formula C_30_H_40_O_11_ with 11 unsaturation degrees, as determined from HRESIMS at *m/z* [M+Na]^+^ 583.2517. The ^1^H and ^13^C NMR data of **3** revealed a similar dimeric structural feature as that of **2**, except for the presence of two extra olefinic carbons (*δ*_C_ 125.3, 164.7) in **3**, and the absence of a methine and an oxygenated quaternary carbon in **2**. Two olefinic carbons were assigned at C-5′ and C-6′ by key HMBC corrections of H-12′ with C-5′, H_2_-8′ with C-5′ and C-6′, H_2_-7′ with C-5′ and C-6′. Further analysis of 2D NMR data (Figs. 2, 4) confirmed the structure of **3**.

All isolated compounds were tested for antibacterial activities against *Staphylococcus aureus* and *Escherichia coli*. None of them showed antibacterial activity at 100 μM.

**Fig. 5:**
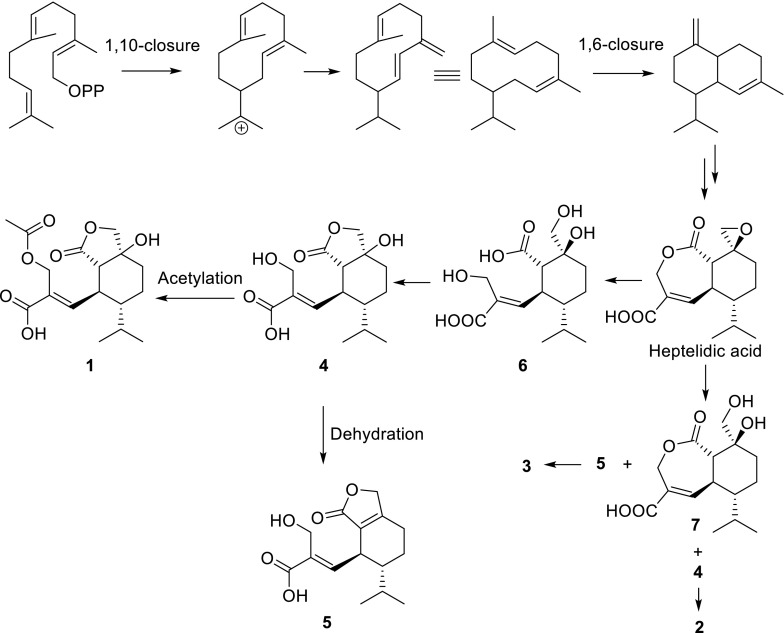
The plausible biosynthetic pathway for compounds **1**–**5**

The sesquiterpene lactone of compounds **1**–**5** are structurally related to each other and might be originated from heptelidic acid. A proposed biogenetic pathway for these compounds is shown in Fig. 5. Heptelidic acid derived from the 1,10- and 1,6-cyclization of FPP [15] was converted into **6** by hydrolysis and oxidation cleavage. **4** was formed from **6** by dehydration, and further transformed into **1** and **5** by acetylation or dehydration, respectively. The intermidate **7** derived from heptelidic acid was further reacted with **4** and **5** to give new dimeric compounds **2** and **3**, respectively.

In conclusion, three previously undescribed cadinane-type sesquiterpenes including one acetylated heptelidic acid derivative (**1**), two dimeric esters (**2**–**3**), and two known heptelidic acid analogues (**4**–**5**), were isolated from the solid culture of *L. ursinus*. The current study enriches the secondary metabolites from this mushroom.

## Experimental

### General Experimental Procedures

HPLC separation was conducted on Agilent 1200 HPLC system equipped with Agilent G1315D DAD detector, using a YMC-Pack ODS-A column (5 μm; 9.4 × 250 mm). NMR spectra were measured on a Bruker Avance-500 spectrometer using solvent signals (CD_3_OD, *δ*_C_/*δ*_H_ 49.00/3.31; CDCl_3_, *δ*_C_/*δ*_H_ 77.16/7.26, DMSO-*d*_6_, *δ*_C_/*δ*_H_ 39.52/2.50) as references. Mass spectra were obtained on an Agilent Accurate-Mass-Q-TOF LC/MS 6520 spectrometer. Optical rotations were recorded on a polarimeter with sodium light (589 nm) by using a Perkin-Elmer 241 polarimeter. UV and IR spectra were recorded on a Thermo Genesys-10S UV–Vis spectrophotometer and a Nicolet IS5FT-IR spectrophotometer, respectively.

### Fungal Material

The strain of *L. ursinus* was isolated from the fruiting bodies of mushroom *L. ursinus* collected in Meilixueshan (Yunnan, China) by Junjie Han, and identified on the basis of the morphological characteristics and ITS sequences. The strain was cultured on PDA plates at 25 °C for 14 days. Agar plugs were inoculated into a 250 mL Erlenmeyer flask containing 100 mL PDB medium cultured at 25 °C on a rotary shaker at 180 rpm for 14 days. Large-scale cultivation was carried out in 20 × 500 mL Fernbach culture flasks each containing 80 g of rice and 100 mL of distilled water. Each flask was inoculated with 5 mL of culture medium and incubated at 25 °C for 40 days in dark.

### Extraction and Isolation

The cultivated rice substrate was extracted repeatedly with EtOAc (3 × 10 L) at room temperature and 20.2 g crude extract was obtained by evaporating solvent under vacuum. The crude extract was subjected to silica gel column chromatography (CC) using CH_2_Cl_2_–MeOH (100:1, 80:1, 60:1, 40:1, 25:1, 10:1, 5:1, 0:1) to give fifteen fractions (Fr.1–Fr.15).

The fraction Fr.7 (4.0 g) eluted with 40:1 CH_2_Cl_2_–MeOH was separated by ODS CC eluting with MeOH–H_2_O gradient elution to give nineteen subfractions (Fr.7.1–Fr.7.19). The fraction Fr.7.4 (1.0 g) was purified by Sephadex LH20 CC eluted with 80% MeOH–H_2_O, followed by PR-HPLC (20% MeCN in H_2_O) to give **4** (10.0 mg, *t*_R_ = 37.8 min). The fraction Fr.7.6 (200.0 mg) was purified by PR-HPLC (28% MeCN in H_2_O) to afford **1** (10.8 mg, *t*_R_ = 26.2 min) and **5** (13.3 mg, *t*_R_ = 30.0 min). Compound **2** (3.5 mg, *t*_R_ = 18.5 min) and **3** (6.2 mg, *t*_R_ = 22.4 min) were purified by PR-HPLC from Fr.7.11 (45% MeCN in H_2_O) and Fr.7.13 (50% MeCN in H_2_O), respectively.

### Spectroscopic Data

#### 3-*O*-Acetylheptelidic acid A (**1**)

Colorless oil; [*α*]_D_^25^ 62.9 (*c* 0.1 MeOH); UV (MeOH) *λ*_max_ (log *ε*) 222 (3.3) nm; IR(neat) *v*_max_ 3432, 2959, 1778, 1719, 1465, 1310, 1233, 1170, 1019 cm^−1^; positive HRESIMS *m/z* [M+Na]^+^ 363.1412 (calcd for C_17_H_24_O_7_Na, 363.1414).

#### Lentisinic acid A (**2**)

Light yellow powder; $[\upalpha ]_{{\text{D}}}^{{25}}$ 23.0 (*c* 0.1 MeOH); UV (MeOH) *λ*_max_ (log *ε*) 217 (3.4) nm; IR(neat) *v*_max_ 3447, 2958, 1734, 1717, 1472, 1388, 1238, 1170, 1018 cm^−1^; positive HRESIMS *m/z* [M+Na]^+^ 601.2615 (calcd for C_30_H_42_O_11_Na, 601.2619).

#### Lentisinic acid B (**3**)

Light yellow powder; $[\upalpha ]_{{\text{D}}}^{{25}}$ 71.5 (*c* 0.2 MeOH); UV (MeOH) *λ*_max_ (log *ε*) 222 (3.4) nm; IR(neat) *v*_max_ 3432, 2959, 1778, 1719, 1465, 1370, 1233, 1170, 1019 cm^−1^; positive HRESIMS *m/z* [M+Na]^+^ 583.2517 (calcd for C_30_H_40_O_11_Na, 583.2514).

### Antimicrobial Assay

The antimicrobial assay was conducted as our previous described method [16]. The bacterial strains *S. aureus* (ATCC 6538) and *E. coli* (ATCC 25922) were grown in Lysogeny Broth (LB) medium. The inhibition rate was calculated and plotted versus test concentrations to afford the MIC. MIC values were defined as the minimum concentration of compound that inhibited visible microbial growth.

## Electronic supplementary material

Below is the link to the electronic supplementary material.

Electronic supplementary material 1 (DOCX 1752 kb)
