# Supplementary material for: Three New Heptelidic Acid Derivatives from the Culture of Mushroom Lentinellus ursinus
Source: Nat Prod Bioprospect. 2018 May 22;8(5):355–60. doi: 10.1007/s13659-018-0168-8 (PMC6109445; doi:10.1007/s13659-018-0168-8)
Supplement: Supplementary file 1 — Electronic supplementary material 1 (DOCX 1752 kb) [file 13659_2018_168_MOESM1_ESM.docx]

**Three new Heptelidic acid Derivatives from the Culture of Mushroom *Lentinellus ursinus***

Li Liu,^1,2^ Jun-jie Han,^1,2^ Tian-shun Xu,^3^ Rui-xing Liu,^1,2^ Li Bao,^1^ Hong-wei Liu^1,2,*^

^1^State Key Laboratory of Mycology, Institute of Microbiology, Chinese Academy of Sciences, Beijing, 100101, People’s Republic of China

^2^Savaid Medical School, University of Chinese Academy of Sciences, Beijing, 100049, People’s Republic of China

^3^College of Life Sciences, Hebei University, Baoding 071002, China

^*^Corresponding Author Tel: +86 10 64806074; E-mail: [liuhw@im.ac.cn](mailto:liuhw@im.ac.cn) (H−W, Liu)

**Table of Contents**

| No. | Contents | Page |
| --- | --- | --- |
| **Figure S1** | ^1^H NMR spectrum of 3-*O*-Acetylheptelidic acid A (**1**) in CD_3_OD (500MHz) | S3 |
| **Figure S2** | ^13^C NMR spectrum of 3-*O*-Acetylheptelidic acid A (**1**) in CD_3_OD (125MHz) | S4 |
| **Figure S3** | ^1^H-^1^H COSY spectrum of 3-*O*-Acetylheptelidic acid A (**1**) in CD_3_OD | S5 |
| **Figure S4** | HSQC spectrum of 3-*O*-Acetylheptelidic acid A (**1**) in CD_3_OD | S6 |
| **Figure S5** | HMBC spectrum of 3-*O*-Acetylheptelidic acid A (**1**) in CD_3_OD | S7 |
| **Figure S6** | ROESY spectrum of 3-*O*-Acetylheptelidic acid A (**1**) in CD_3_OD | S8 |
| **Figure S7** | HRESIMS spectrum of 3-*O*-Acetylheptelidic acid A (**1**) | S9 |
| **Figure S8** | ^1^H NMR spectrum of 3-*O*-Acetylheptelidic acid A (**1**) in DMSO-*d*_6_ (500MHz) | S10 |
| **Figure S9** | ROESY spectrum of 3-*O*-Acetylheptelidic acid A (**1**) in DMSO-*d*_6_ | S11 |
| **Figure S10** | ^1^H NMR spectrum of Lentisinic acid A (**2**) in CD_3_OD (500MHz) | S12 |
| **Figure S11** | ^13^C NMR spectrum of Lentisinic acid A (**2**) in CD_3_OD (125MHz) | S13 |
| **Figure S12** | ^1^H-^1^H COSY spectrum of Lentisinic acid A (**2**) in CD_3_OD | S14 |
| **Figure S13** | HSQC spectrum of Lentisinic acid A (**2**) in CD_3_OD | S15 |
| **Figure S14** | HMBC spectrum of Lentisinic acid A (**2**) in CD_3_OD | S16 |
| **Figure S15** | ROESY spectrum of Lentisinic acid A (**2**) in CD_3_OD | S17 |
| **Figure S16** | HRESIMS spectrum of Lentisinic acid A (**2**) | S18 |
| **Figure S17** | ^1^H NMR spectrum of Lentisinic acid B (**3**) in CDCl_3_ (500MHz) | S19 |
| **Figure S18** | ^13^C NMR spectrum of Lentisinic acid B (**3**) in CDCl_3_ (125MHz) | S20 |
| **Figure S19** | ^1^H-^1^H COSY spectrum of Lentisinic acid B (**3**) in CDCl_3_ | S21 |
| **Figure S20** | HSQC spectrum of Lentisinic acid B (**3**) in CDCl_3_ | S22 |
| **Figure S21** | HMBC spectrum of Lentisinic acid B (**3**) in CDCl_3_ | S23 |
| **Figure S22** | ROESY spectrum of Lentisinic acid B (**3**) in CDCl_3_ | S24 |
| **Figure S23** | HRESIMS spectrum of Lentisinic acid B (**3**) | S25 |
| **Figure S24** | ^1^H NMR spectrum of compound **4** in CD_3_OD (500MHz) | S26 |
| **Figure S25** | ^13^C NMR spectrum of compound **4** in CD_3_OD (125MHz) | S27 |
| **Figure S26** | ^1^H NMR spectrum of compound **5** in CD_3_OD (500MHz) | S28 |
| **Figure S27** | ^13^C NMR spectrum of compound **5** in CD_3_OD (125MHz) | S29 |


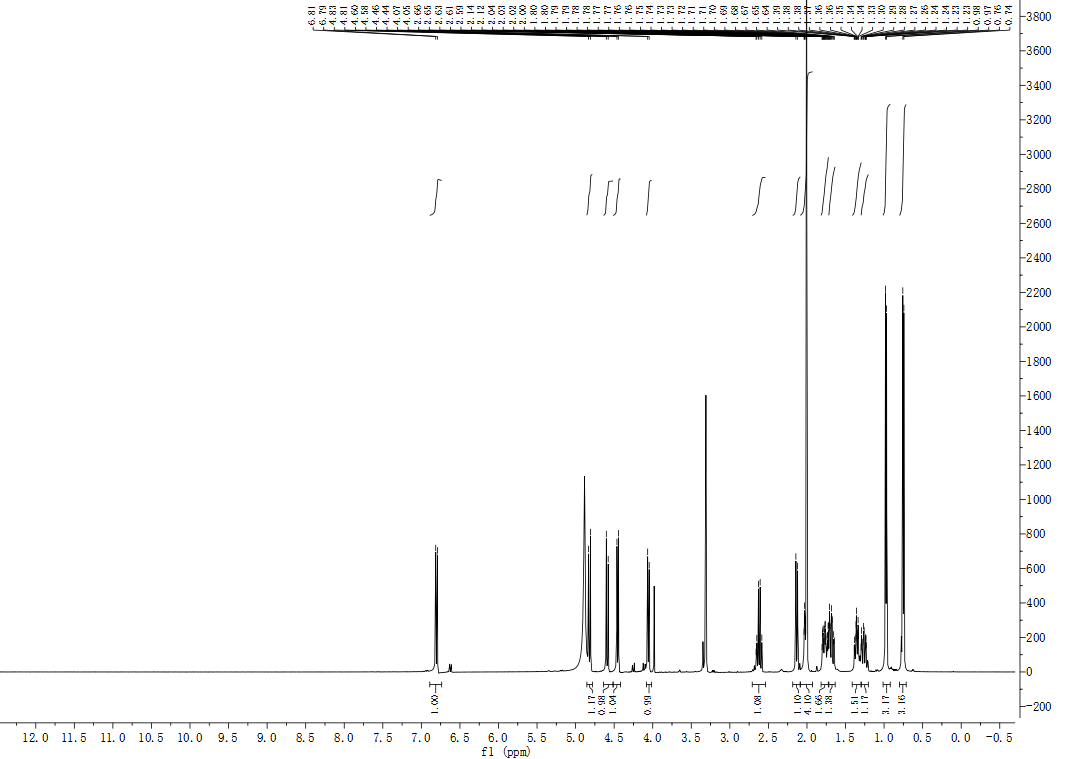


**Figure S1**. ^1^H NMR spectrum of 3-*O*-Acetylheptelidic acid A (**1**) in CD_3_OD (500MHz)


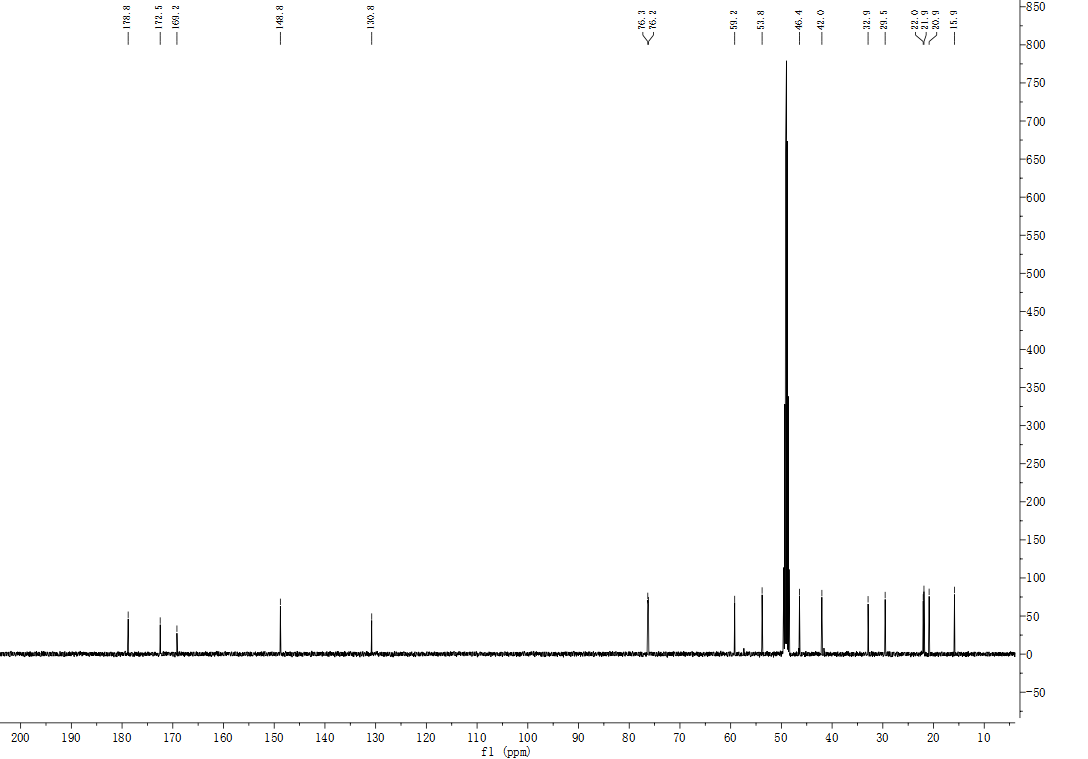


**Figure S2.** ^13^C NMR spectrum of 3-*O*-Acetylheptelidic acid A (**1**) in CD_3_OD (125MHz)

**
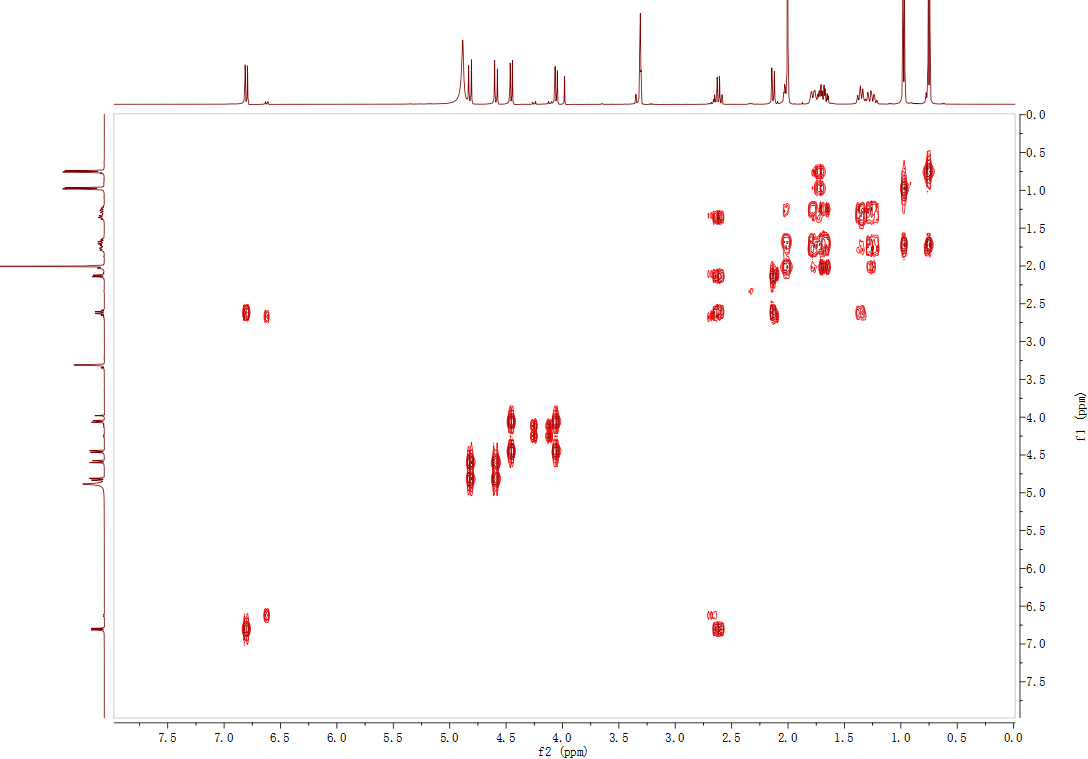
**

**Figure S3.** ^1^H-^1^H COSY spectrum of 3-*O*-Acetylheptelidic acid A (**1**) in CD_3_OD

**
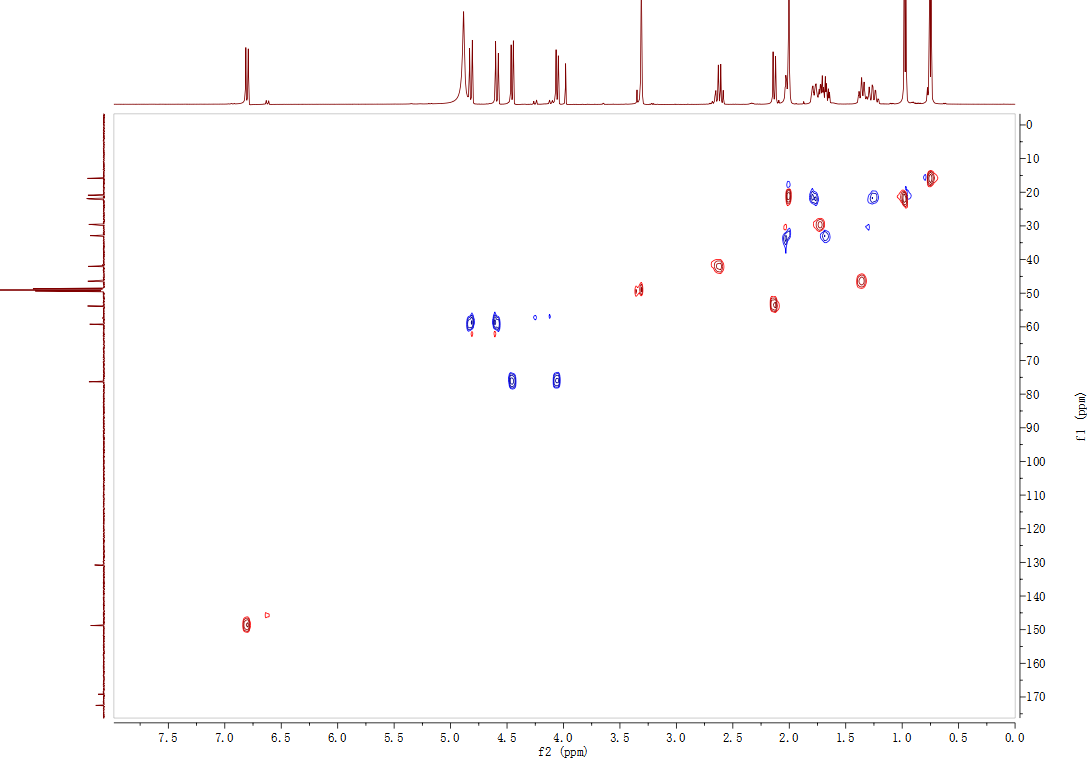
**

**Figure S4.** HSQC spectrum of 3-*O*-Acetylheptelidic acid A (**1**) in CD_3_OD

**
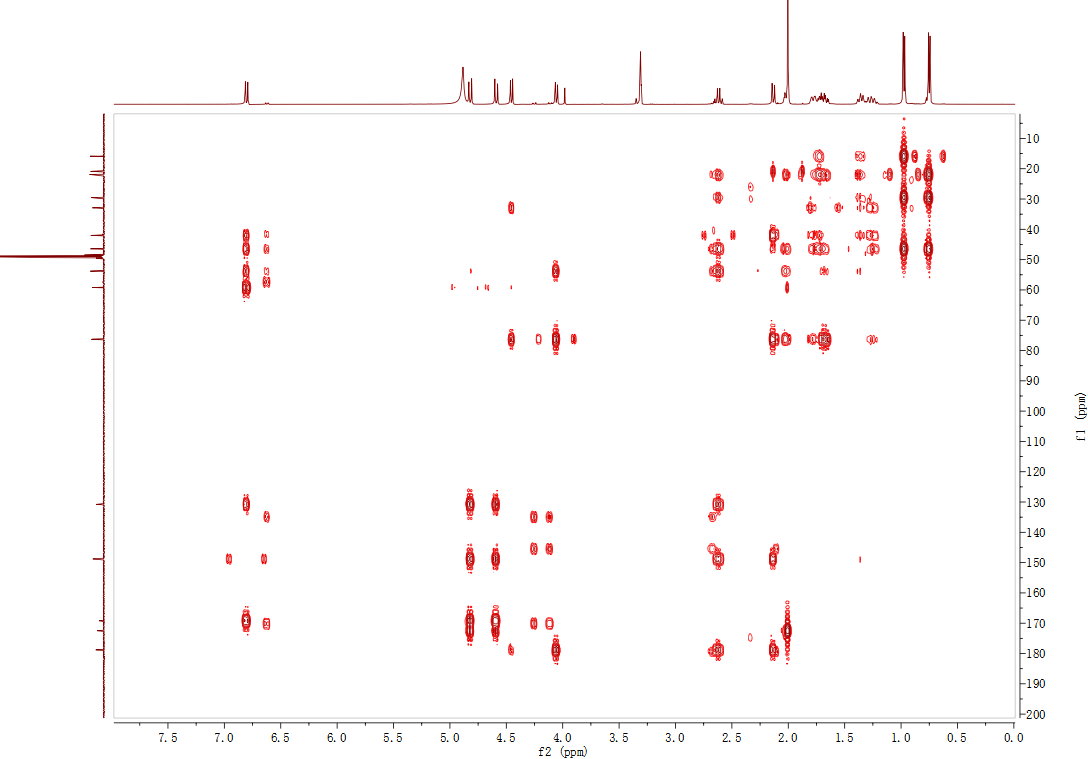
**

**Figure S5.** HMBC spectrum of 3-*O*-Acetylheptelidic acid A (**1**) in CD_3_OD


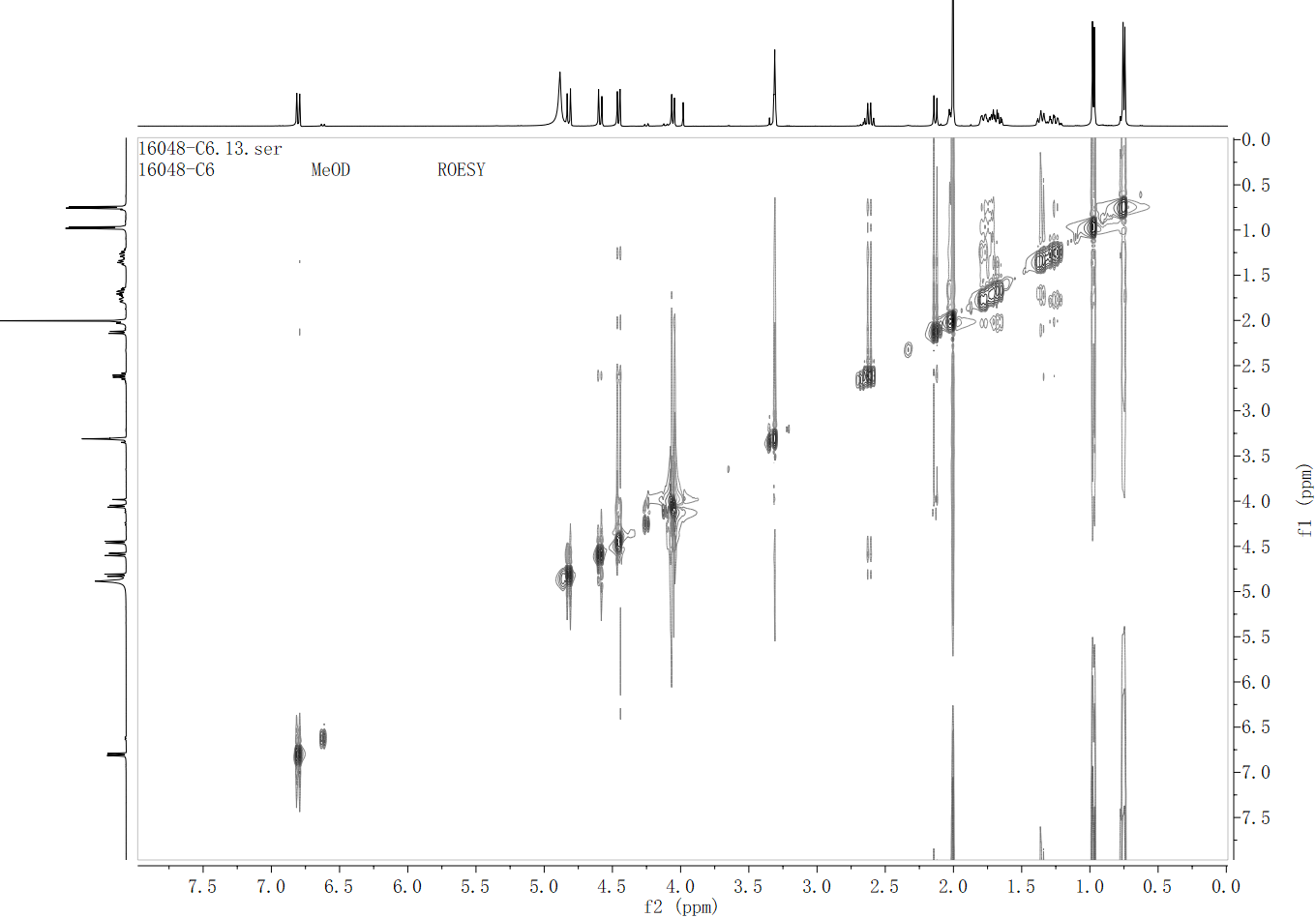


**Figure S6.** ROESY spectrum of 3-*O*-Acetylheptelidic acid A (**1**) in CD_3_OD

**
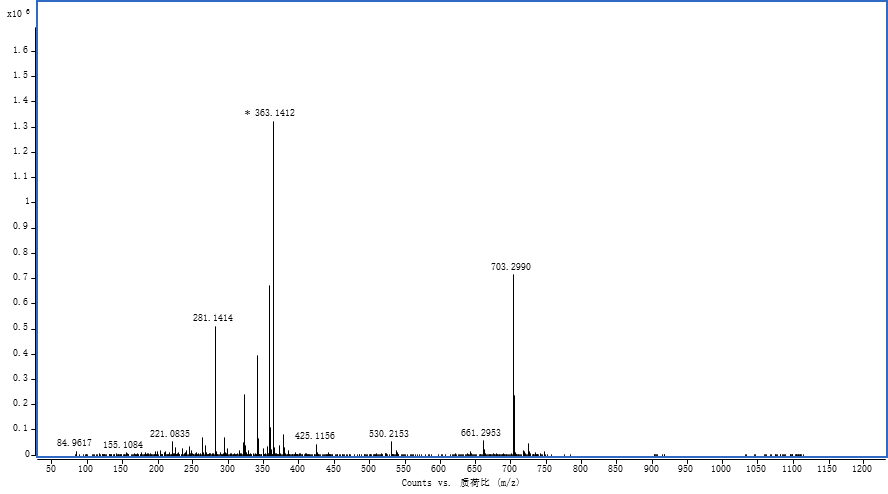
**

**Figure S7.** HRESIMS spectrum of 3-*O*-Acetylheptelidic acid A (**1**)

**
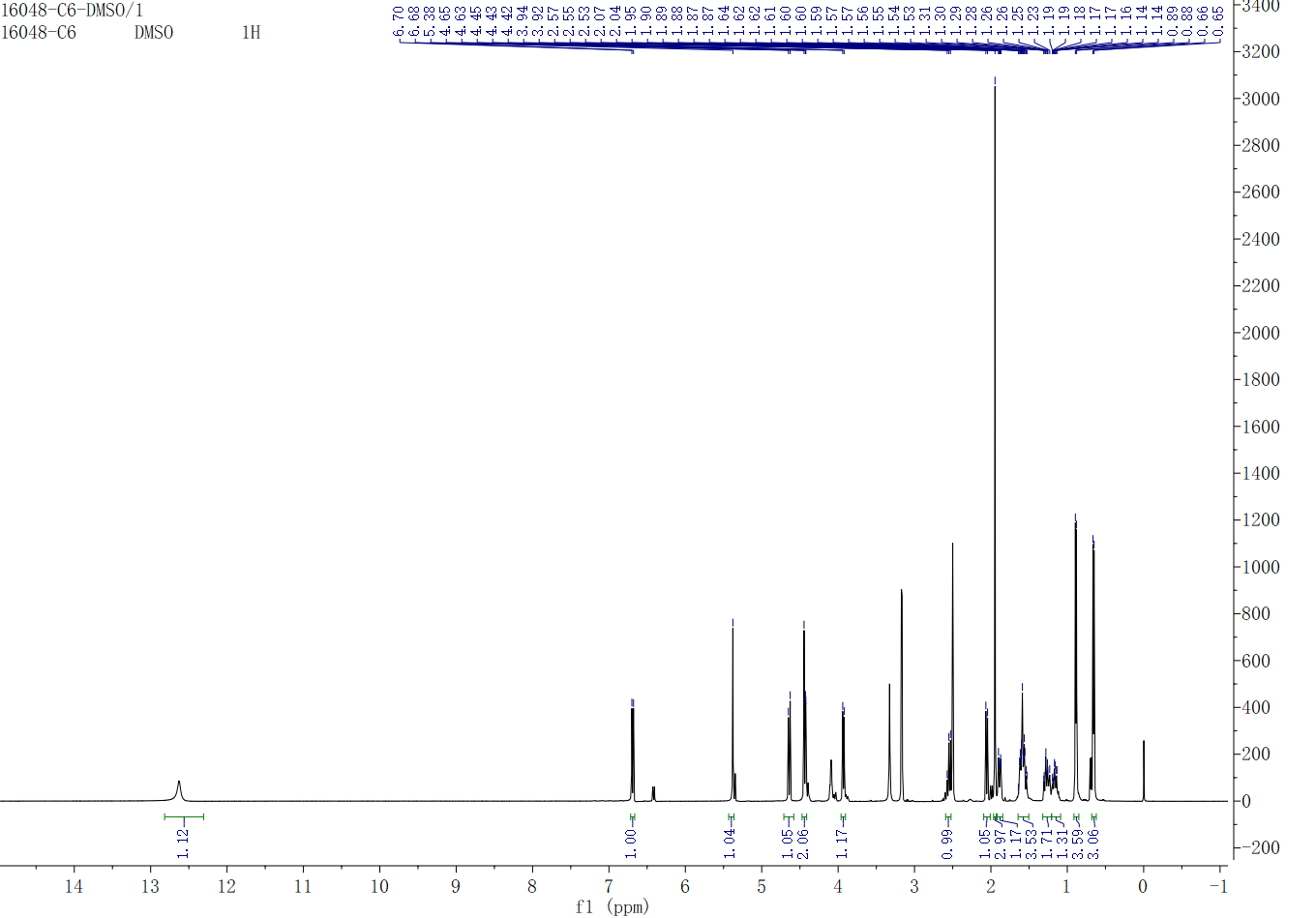
**

**Figure S8.** ^1^H NMR spectrum of 3-*O*-Acetylheptelidic acid A (**1**) in DMSO-*d*_6_ (500MHz)

**
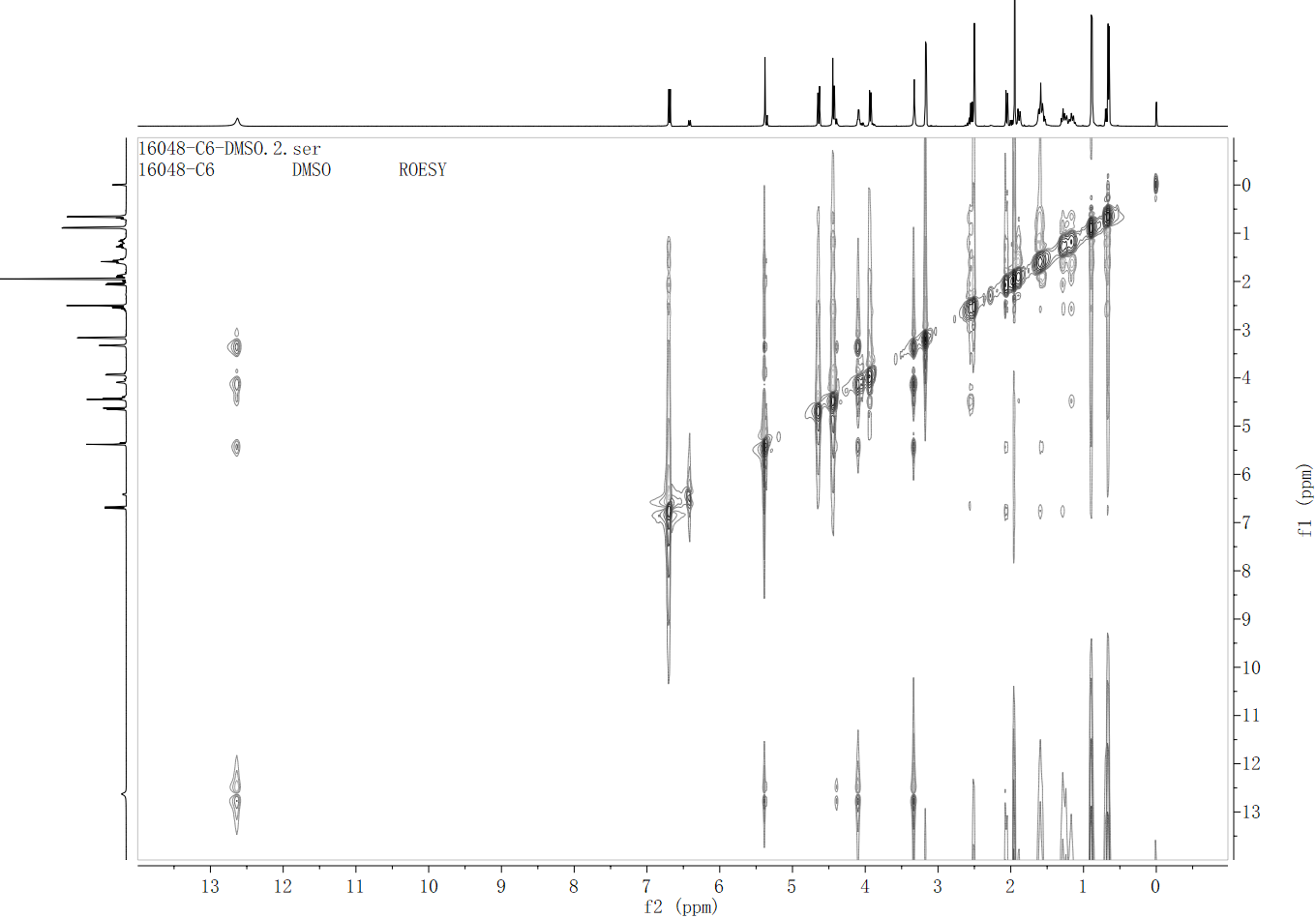
**

**Figure S9.** ROESY spectrum of 3-*O*-Acetylheptelidic acid A (**1**) in DMSO-*d*_6_

**
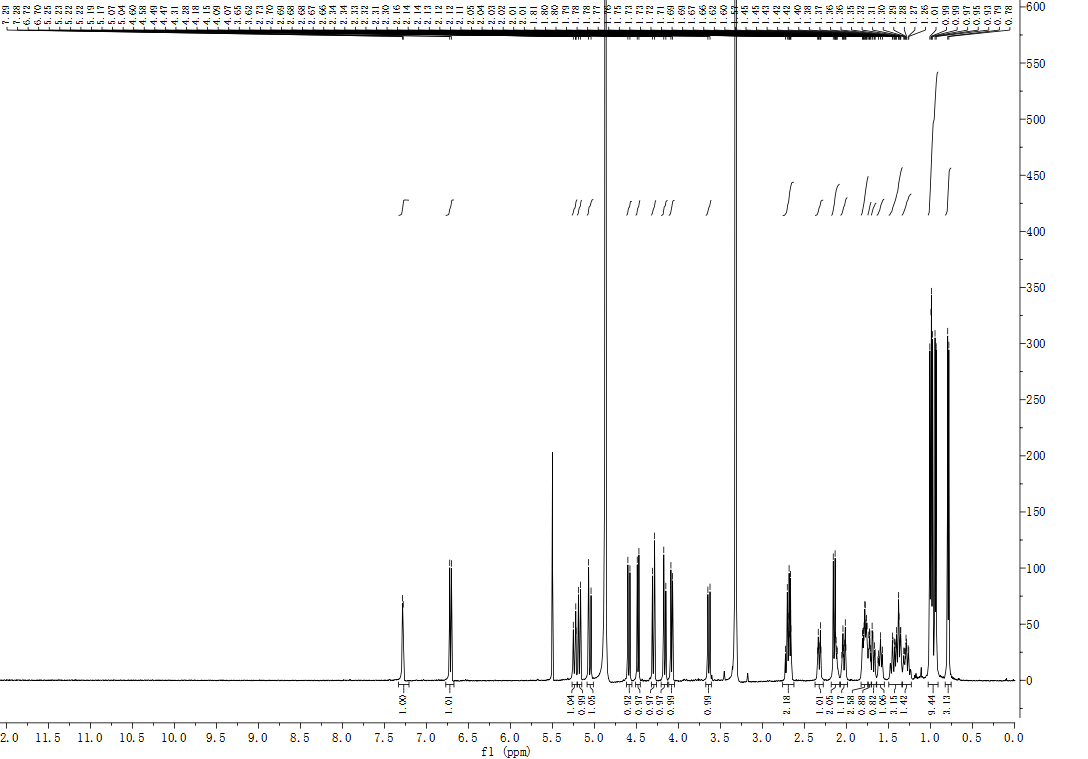
**

**Figure S10.** ^1^H NMR spectrum of Lentisinic acid A (**2**) in CD_3_OD (500MHz)

**
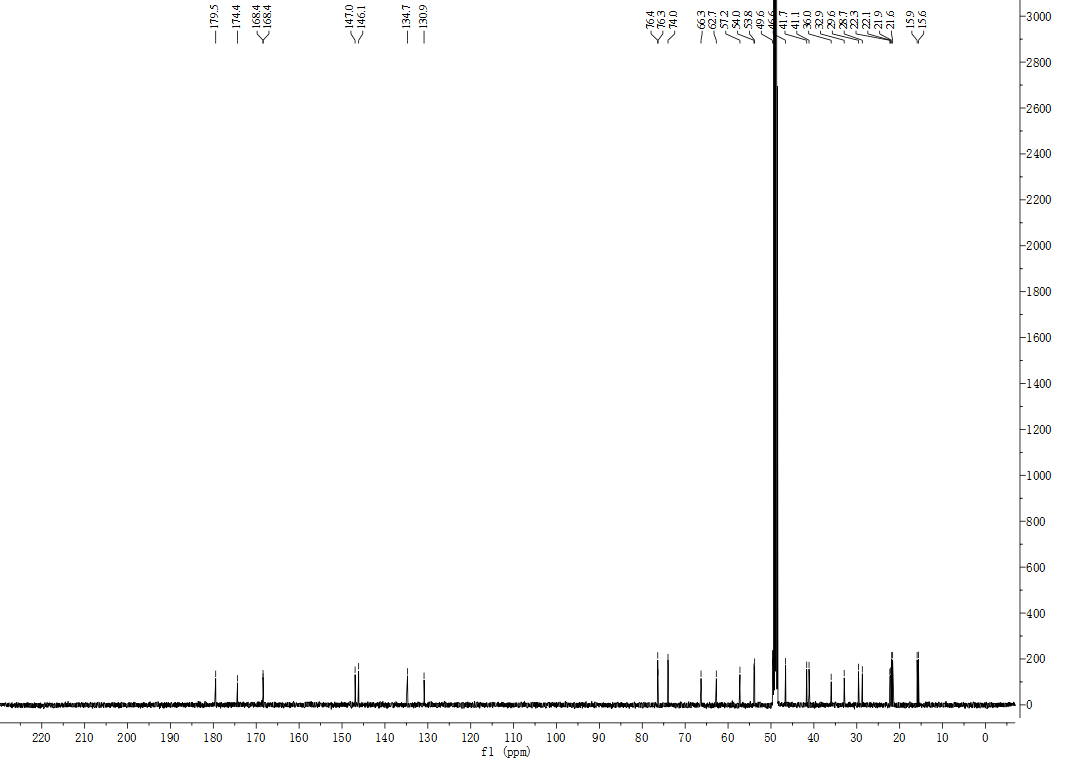
**

**Figure S11.** ^13^C NMR spectrum of Lentisinic acid A (**2**) in CD_3_OD (125MHz)

**
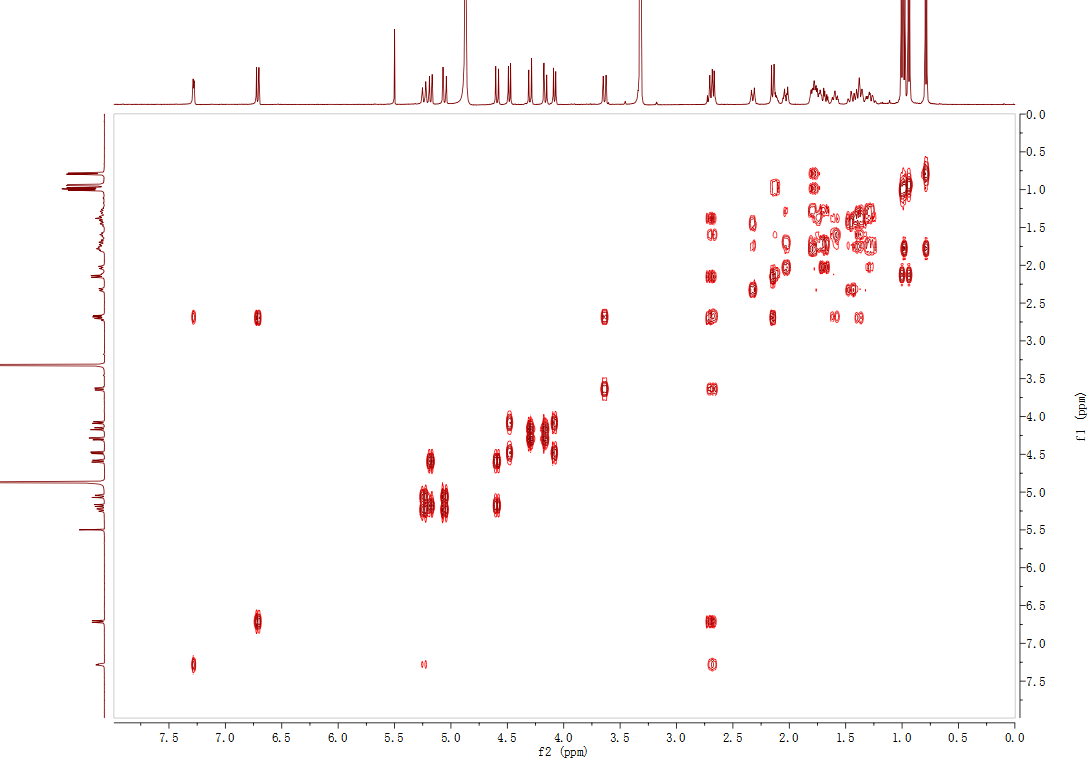
**

**Figure S12.** ^1^H-^1^H COSY spectrum of Lentisinic acid A (**2**) in CD_3_OD

**
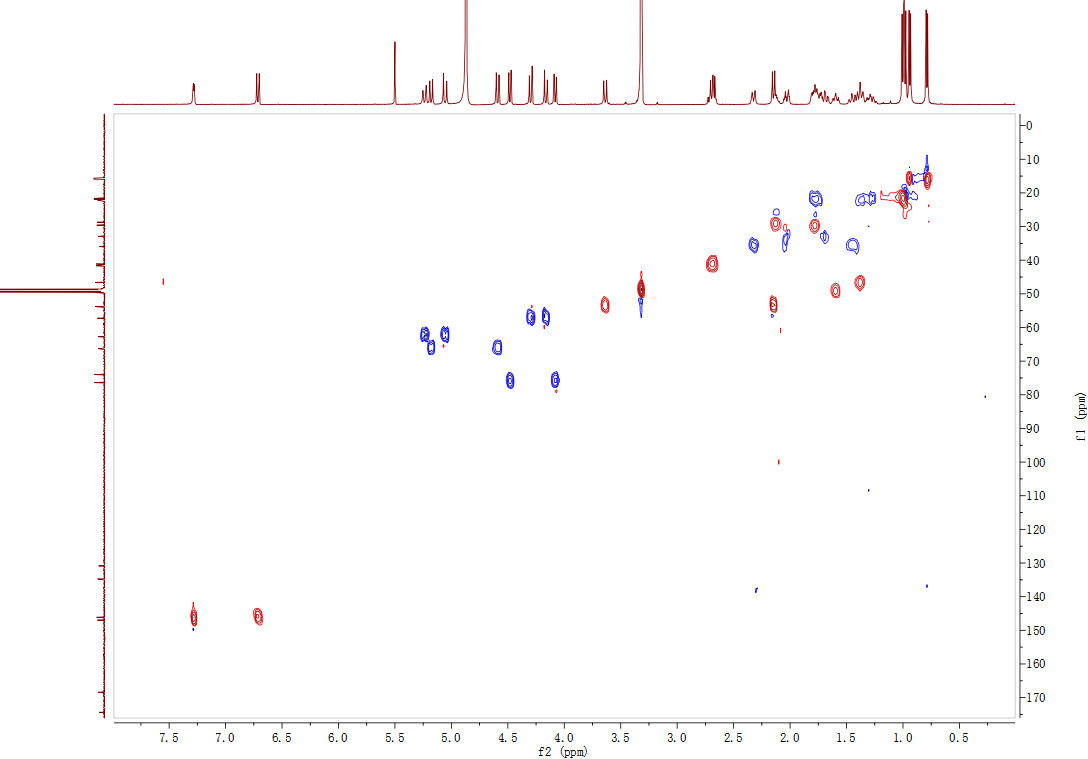
**

**Figure S13.** HSQC spectrum of Lentisinic acid A (**2**) in CD_3_OD

**
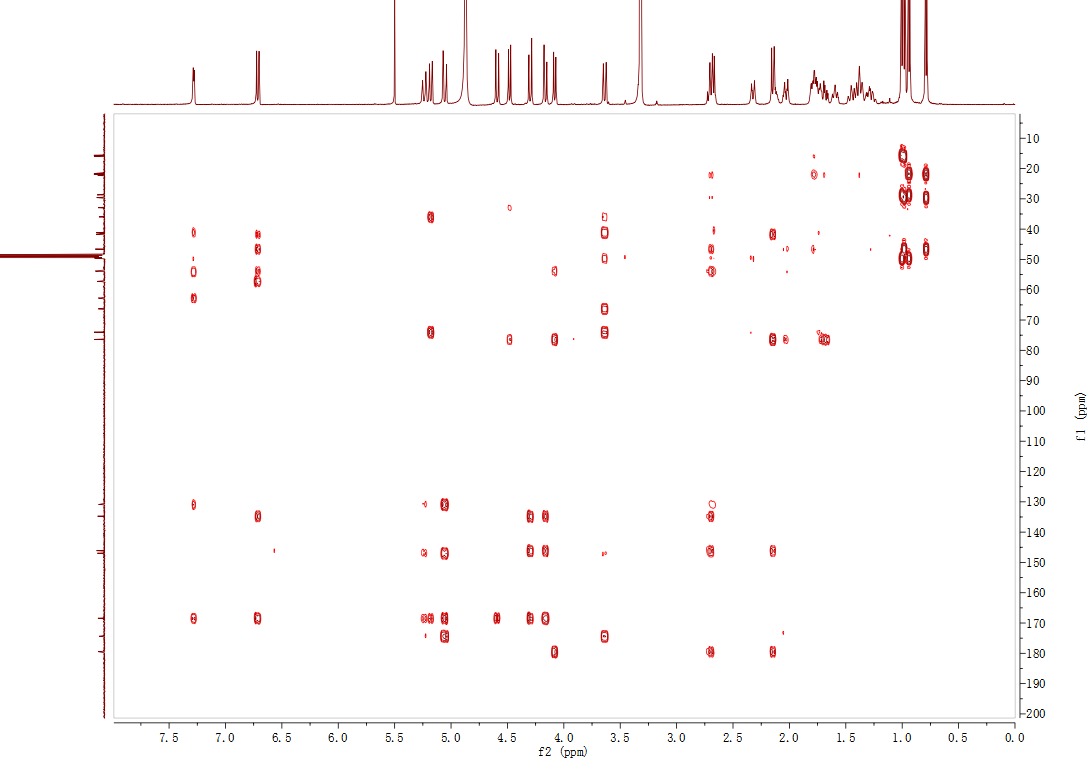
**

**Figure S14.** HSQC spectrum of Lentisinic acid A (**2**) in CD_3_OD


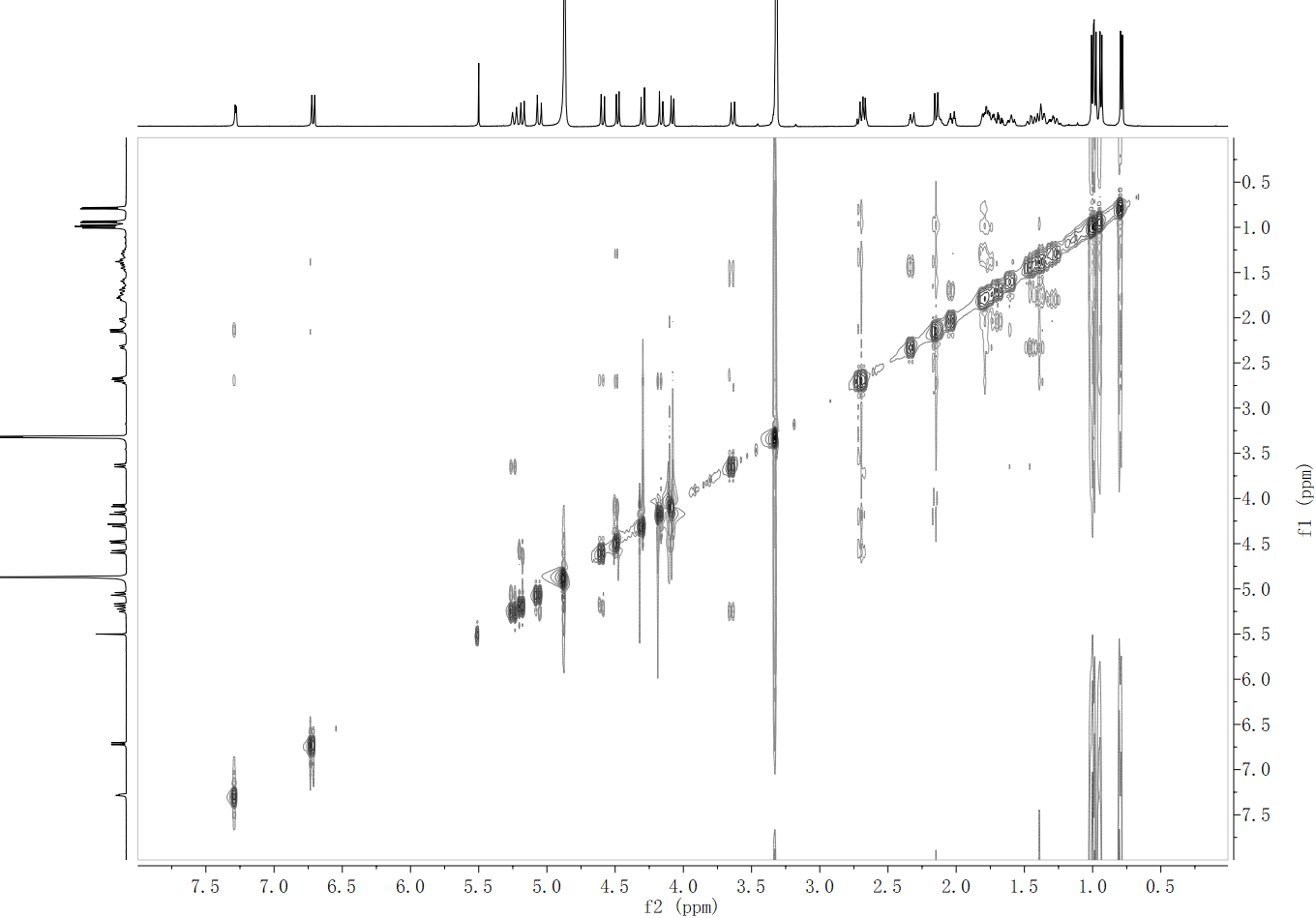


**Figure S15.** ROESY spectrum of Lentisinic acid A (**2**) in CD_3_OD

**
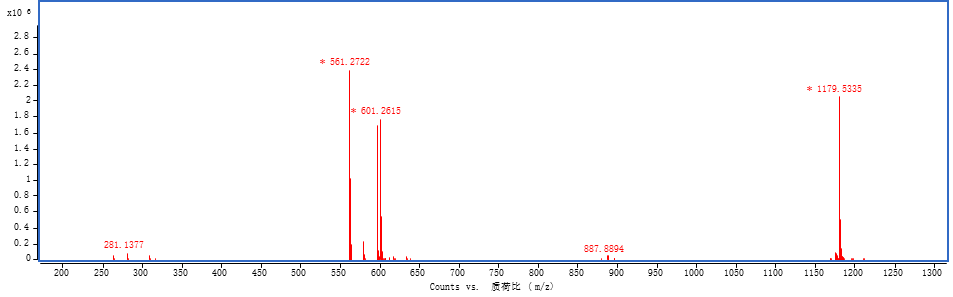
**

**Figure S16.** HRESIMS spectrums of Lentisinic acid A (**2**)

**
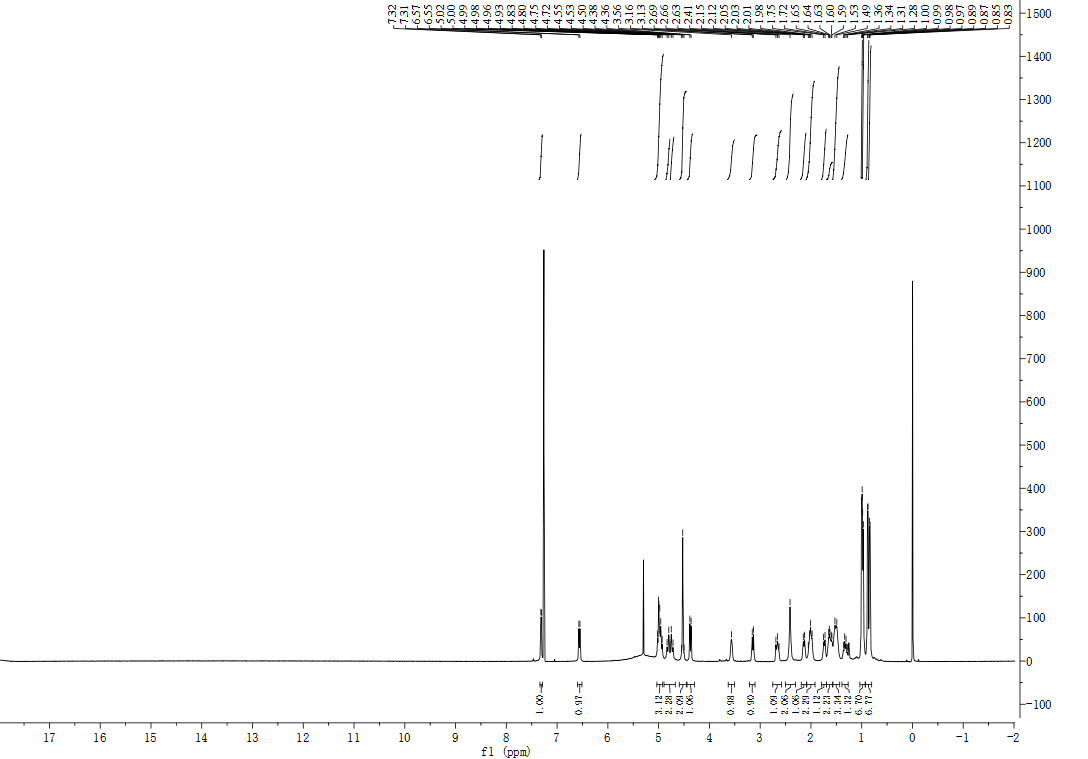
**

**Figure S17.** ^1^H NMR spectrum of Lentisinic acid B (**3**) in CDCl_3_ (500MHz)

**
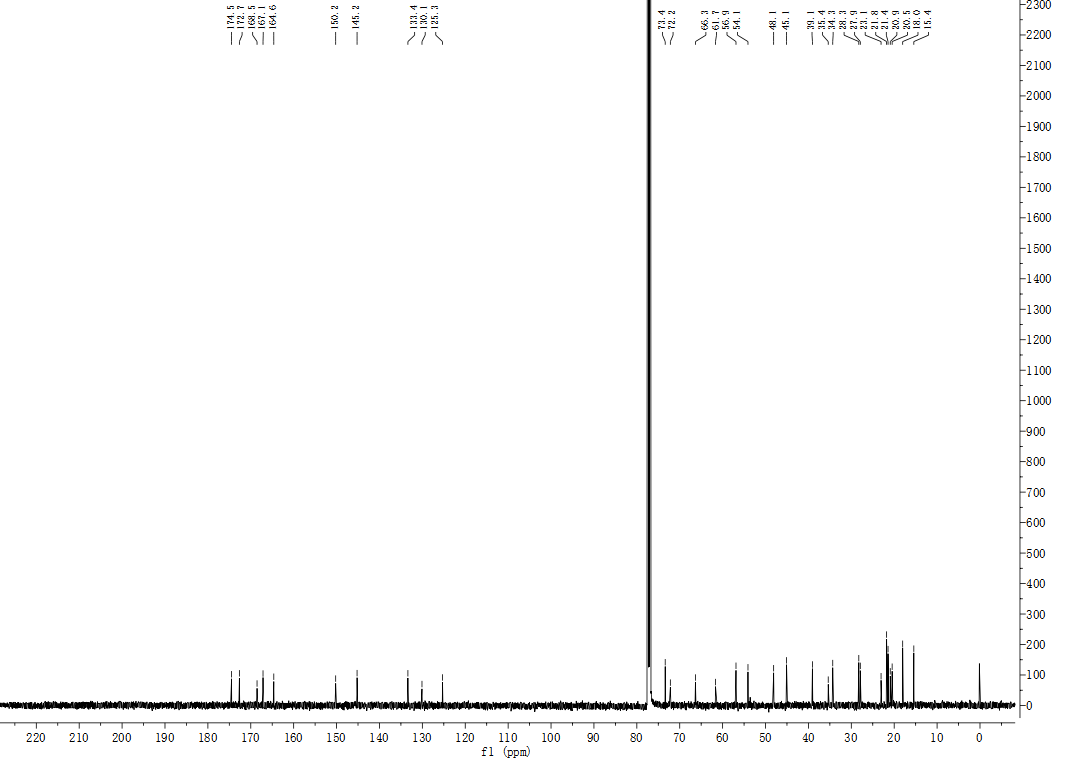
**

**Figure S18.**^13^C NMR spectrum of Lentisinic acid B (**3**) in CDCl_3_ (125MHz)

**
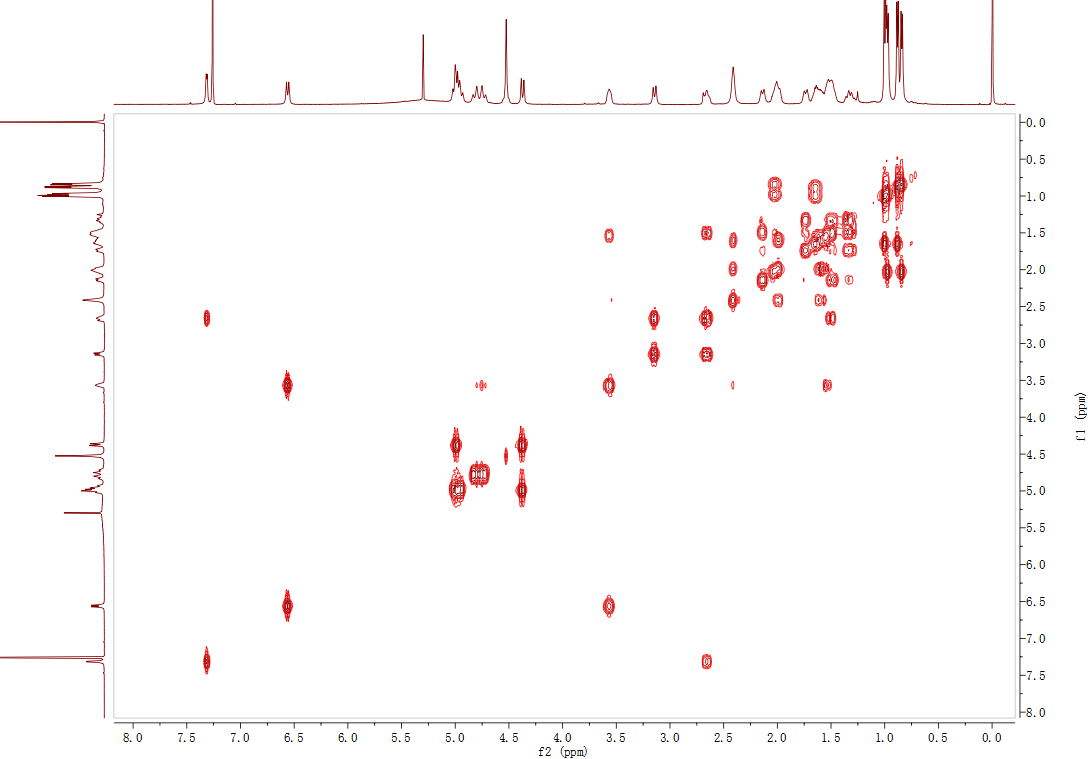
**

**Figure S19.**^1^H-^1^H COSY spectrum of Lentisinic acid B (**3**) in CDCl_3_

**
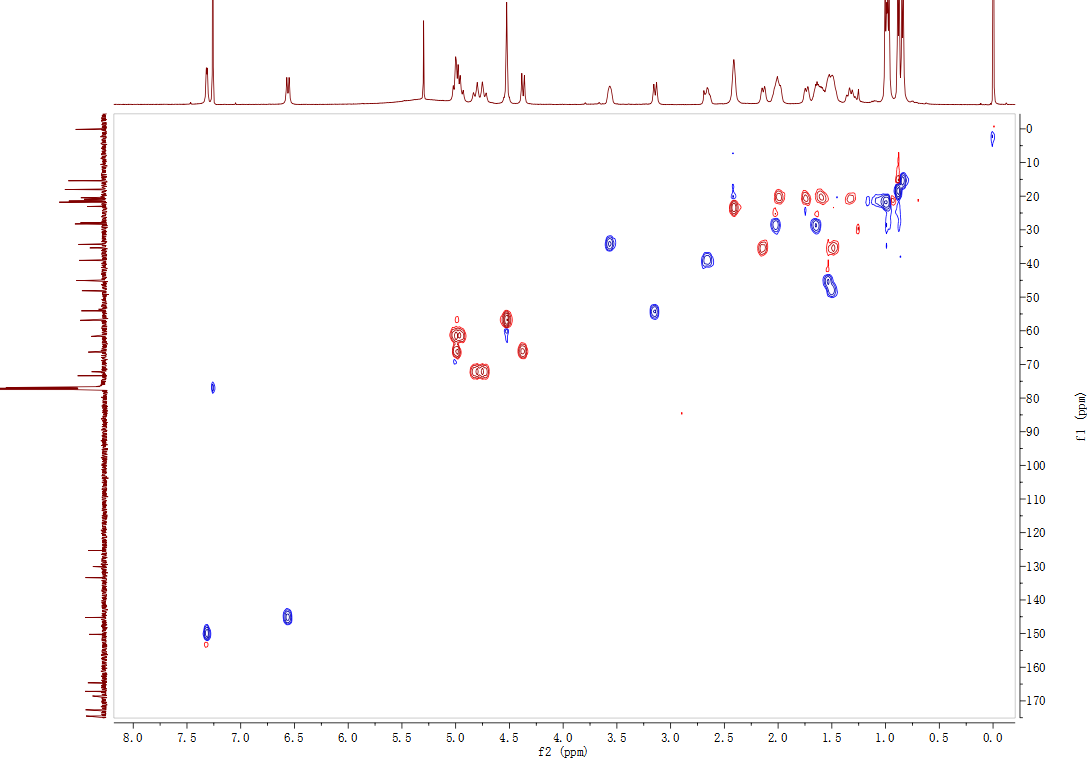
**

**Figure S20.** HSQC spectrum of Lentisinic acid B (**3**) in CDCl_3_

**
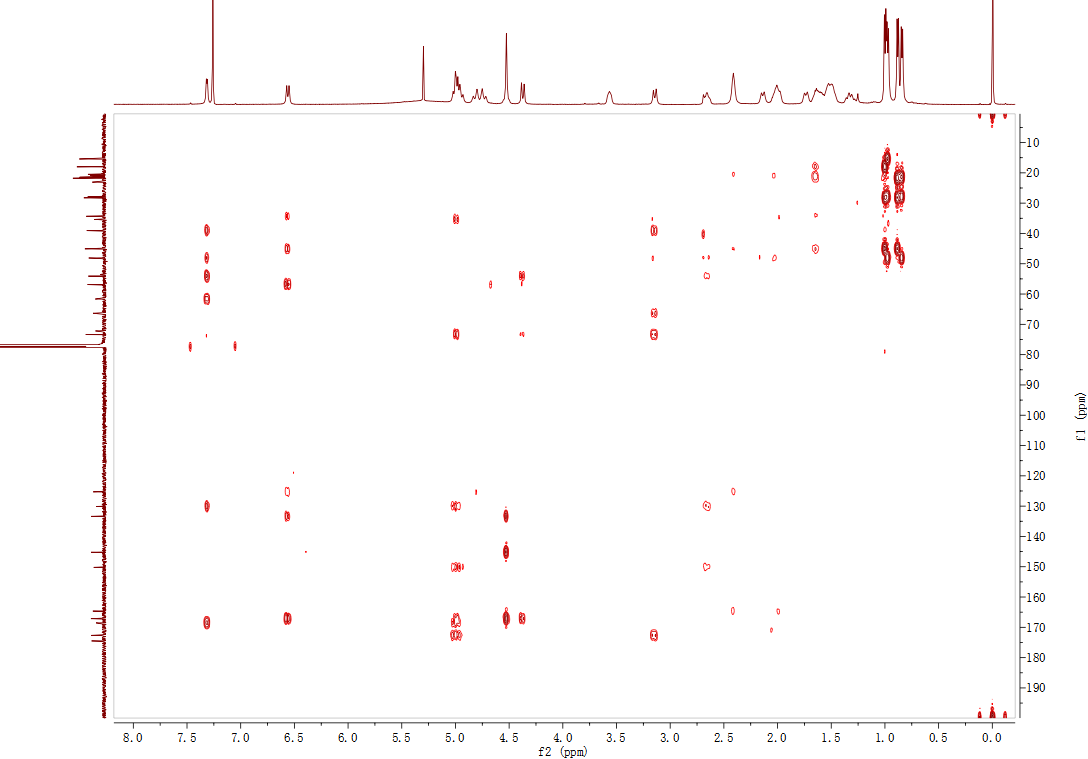
**

**Figure S21.** HMBC spectrum of Lentisinic acid B (**3**) in CDCl_3_


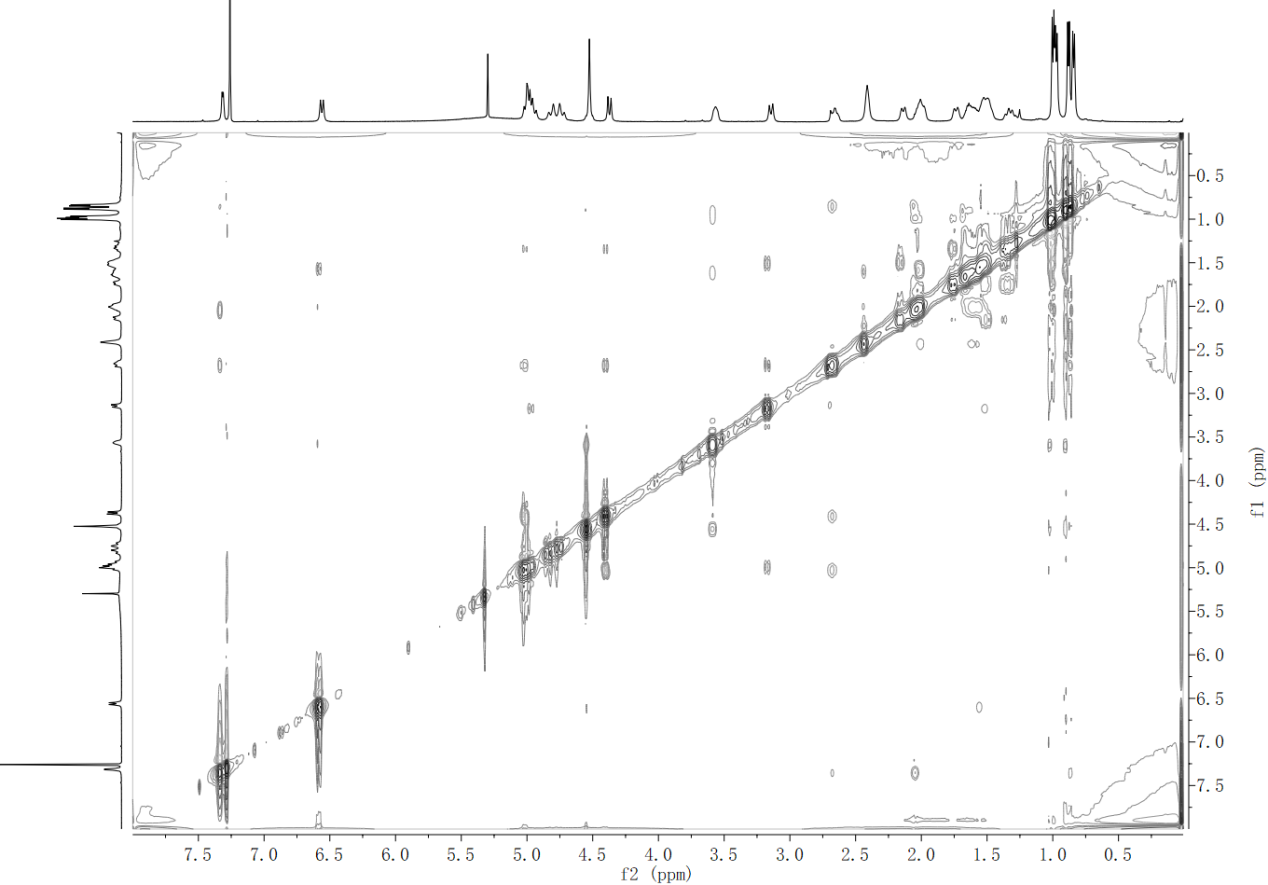


**Figure S22.** ROESY spectrum of Lentisinic acid B (**3**) in CDCl_3_

**
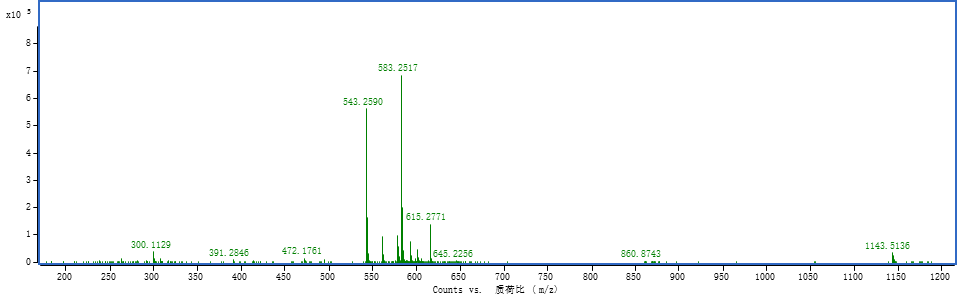
**

**Figure S23.** HRESIMS of Lentisinic acid B (**3**) in CDCl_3_

**
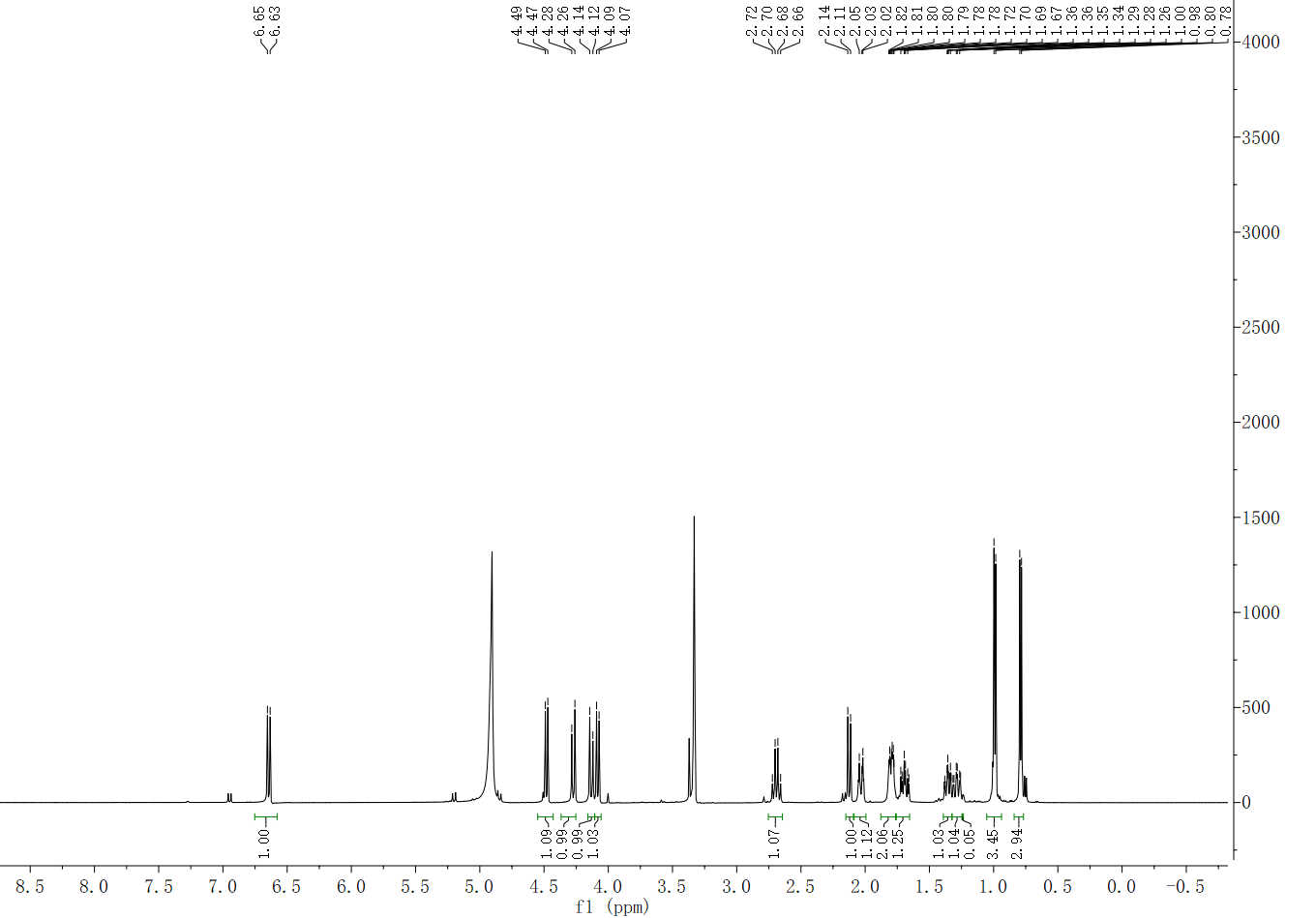
**

**Figure S24.** ^1^H NMR spectrum of compound **4** in CD_3_OD (500MHz)

**
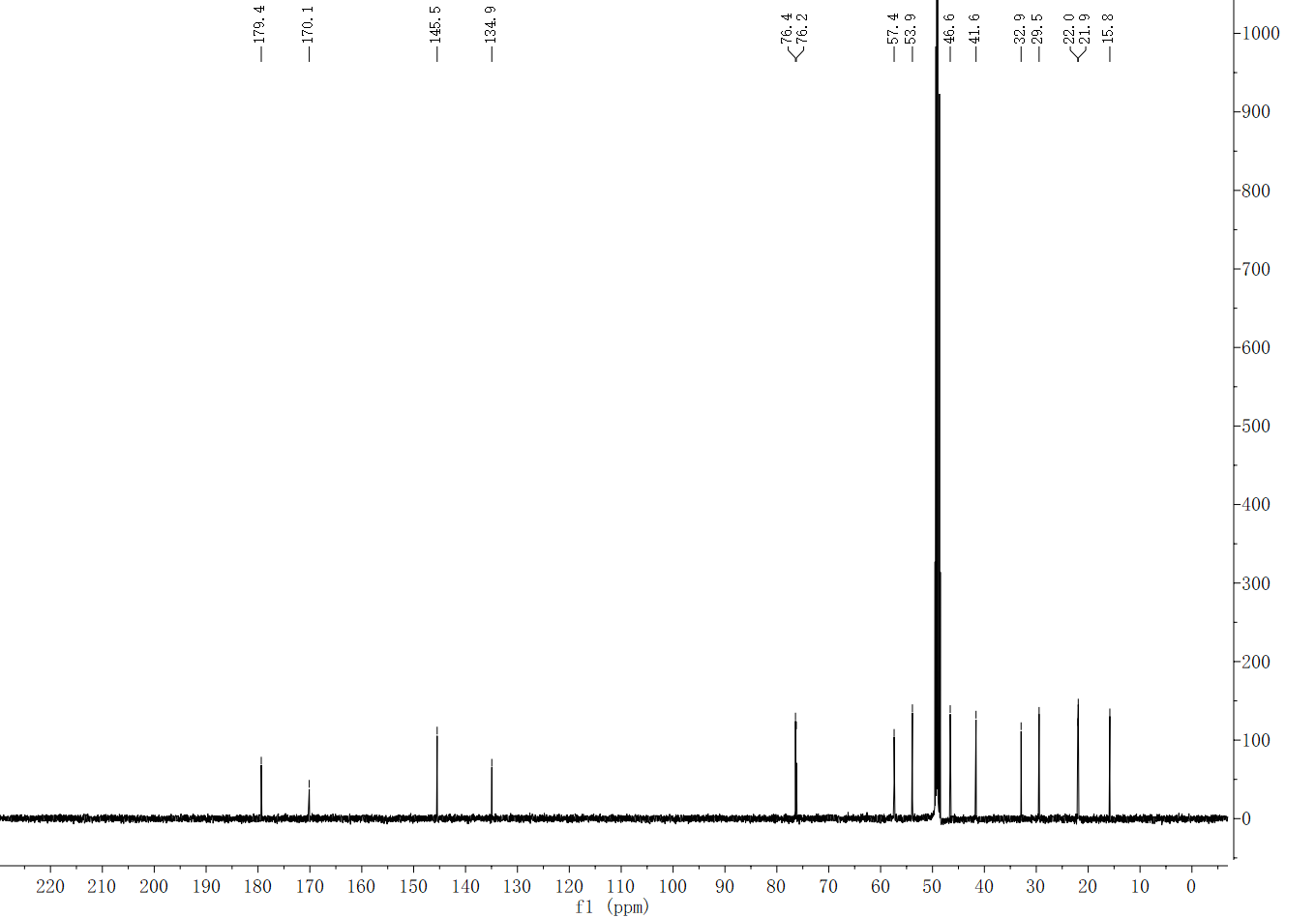
**

**Figure S25.**^13^C NMR spectrum of compound **4** in CD_3_OD (125MHz)

**
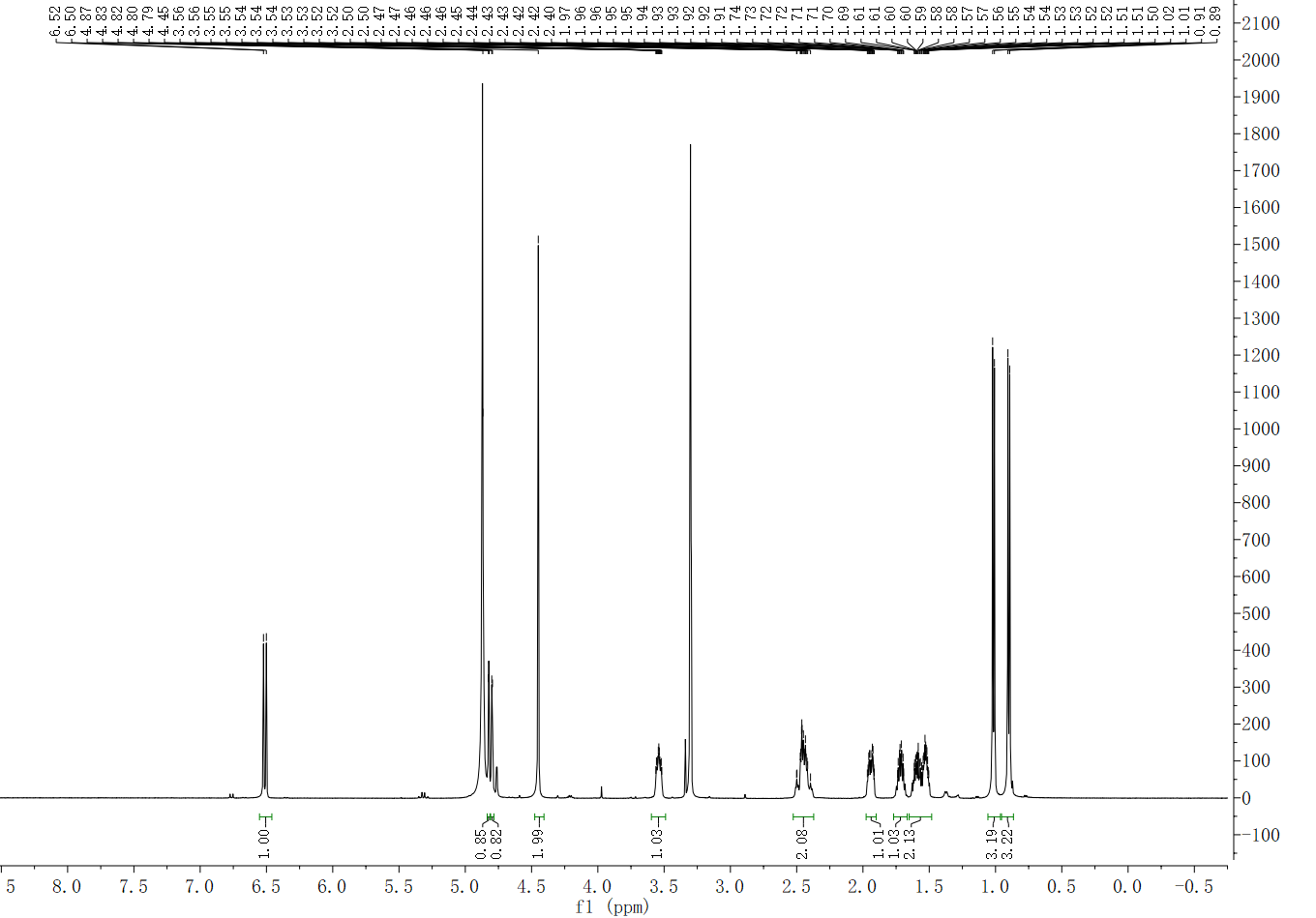
**

**Figure S26.** ^1^H NMR spectrum of compound **5** in CD_3_OD (500MHz)

**
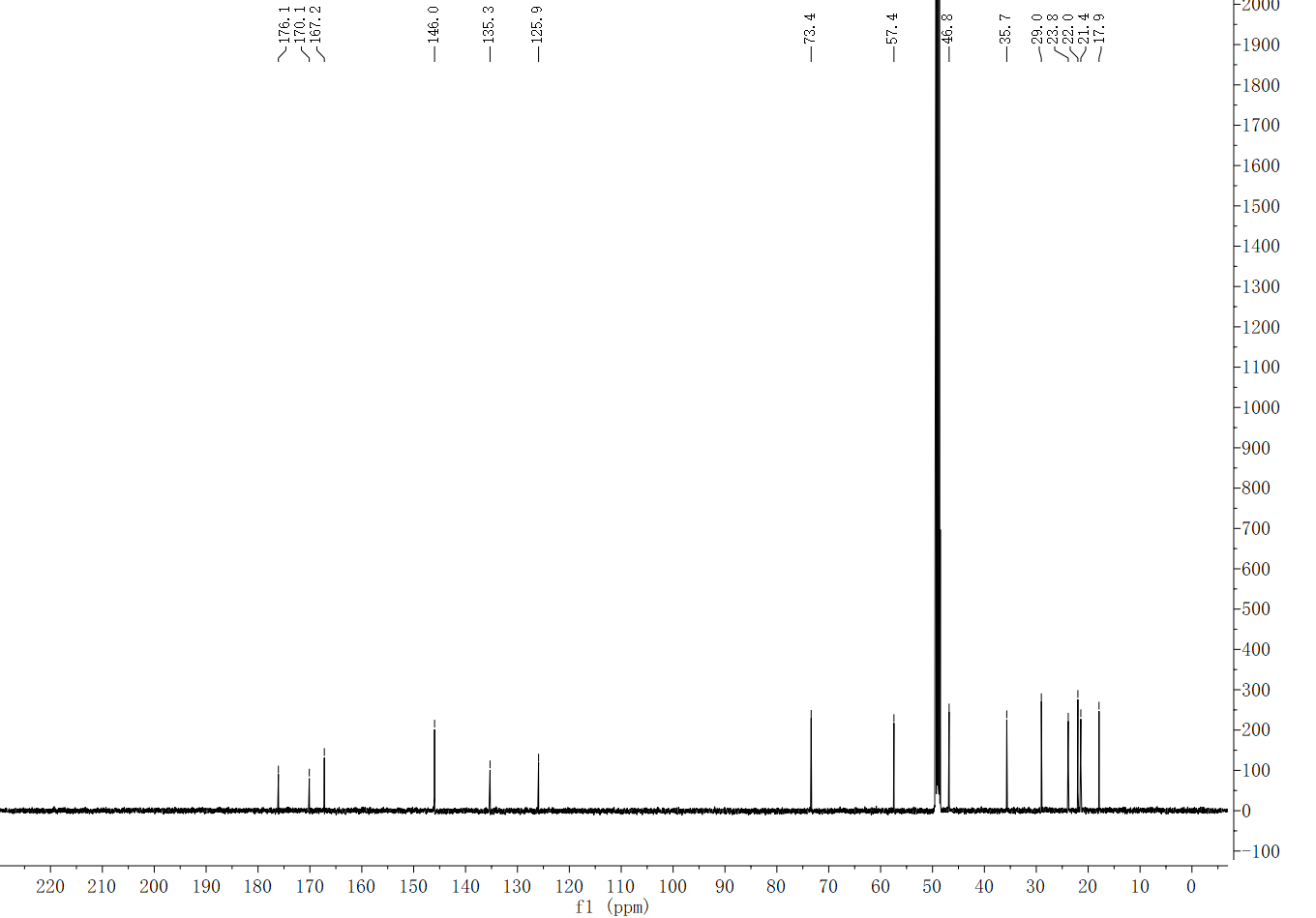
**

**Figure S27.**^13^C NMR spectrum of compound **5** in CD_3_OD (125MHz)
